# NK cell infusion is well-tolerated and shows preliminary efficacy in patients with recurrent hepatocellular carcinoma post-liver transplantation : a phase I trial

**DOI:** 10.1186/s12967-026-07725-x

**Published:** 2026-01-24

**Authors:** Fan Yang, Yihang Gong, Xiaofang Zheng, Beibei Ni, Jianxi Lu, Xiaoyan Chen, Jintao Cheng, Panlong Li, Cong Du, Yunhao Chen, Yingcai Zhang, Shuhong Yi, Guoying Wang, Qi Zhang, Yang Yang, Wenjie Chen

**Affiliations:** 1 Biotherapy Centre, The Third Affiliated Hospital, Sun Yat-Sen University, Guangzhou, 510630 PR China; 2 Xinjiang Stem Cells Special Plateau Disease Engineering Technology Research Center, The First People’s Hospital of Kashi, (The Affiliated Kashi Hospital of Sun Yat-Sen University), Kashi, 844000 PR China; 3 Cell–Gene Therapy Translational Medicine Research Centre, The Third Affiliated Hospital, Sun Yat-Sen University, Guangzhou, 510630 PR China; 4 Department of Hepatic Surgery and Liver Transplantation Centre, The Third Affiliated Hospital, Sun Yat-Sen University, Guangzhou, 510630 PR China; 5 Department of Hepatobiliary Surgery, the First Affiliated Hospital of Guangzhou Medical University, Guangzhou, 510120 PR China

**Keywords:** NK cell infusion, Recurrent, Post-Liver transplantation, Tolerated, Effective

## Abstract

**Background:**

Recurrent hepatocellular carcinoma (HCC) after liver transplantation remains a formidable challenge. This Phase 1, dose-escalation trial had the ​primary objectives​ of evaluating the safety and tolerability of various natural killer (NK) cell infusion regimens in patients with recurrent HCC. ​As exploratory endpoints, the study also assessed preliminary antitumor activity, with progression-free survival (PFS) and overall survival (OS) being key measures of interest.

**Methods:**

Between December 31, 2014, and March 29, 2017, 18 patients with recurrent HCC after liver transplantation were enrolled in this single-center, Phase I dose-exploration study. Patients were allocated to four treatment groups to receive different frequencies and doses of NK cell infusions alongside conventional treatment. Group A (*n* = 3) received four low-dose infusions, Group B (*n* = 5) received four normal-dose infusions, Group C (*n* = 6) received eight normal-dose infusions, and Group D (*n* = 4) followed an incremental dosing schedule. Treatment-related adverse events (AEs) and survival outcomes were systematically evaluated, with a maximum follow-up period of 9 years.

**Results:**

The most common AE was Grade 1 pyrexia, which typically resolved within a day. The median PFS across the cohort was 4.8 months, with significant differences observed among the groups (log-rank *P* = 0.0008). Specifically, the PFS was 1.6 months for Group A, 2.5 months for Group B, 5.5 months for Group C, and 7.5 months for Group D. The median OS was 17.7 months, with notable differences among the groups (log-rank *P* = 0.0403). The OS durations were 12.6 months for Group A, 16.1 months for Group B, 18.4 months for Group C, and 31.3 months for Group D.

**Conclusion:**

NK cell infusions were well tolerated and associated with differences in both PFS and OS in patients with recurrent HCC after liver transplantation. Both the frequency and dosage of NK cell infusions are crucial factors influencing survival outcomes, suggesting a potential dose-frequency response relationship.These findings preliminarily underscore the potential of optimizing NK cell-based immunotherapies to enhance clinical outcomes in this challenging patient population.

**Trial registration:**

Trial registration NCT, NCT02399735, Registered 23 March 2015 - Retrospectively registered, https://clinicaltrials.gov/study/NCT02399735.

**Graphical Abstract:**

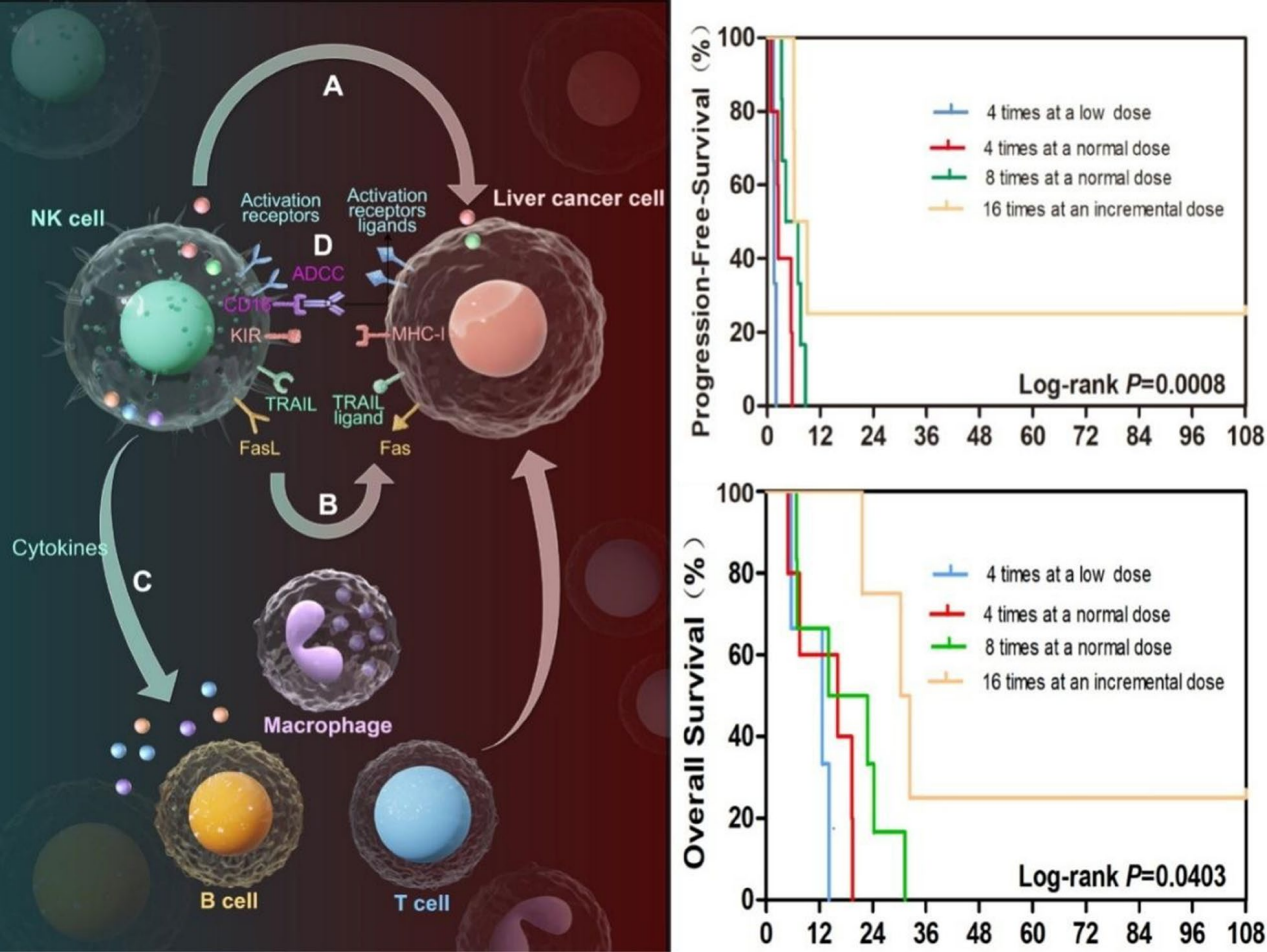

**Supplementary Information:**

The online version contains supplementary material available at 10.1186/s12967-026-07725-x.

## Background

Liver cancers primarily originate in the liver parenchyma or bile ducts, with hepatocellular carcinoma (HCC) being the predominant type. It accounts for more than 90% of primary liver cancer cases [1]. The incidence of HCC has been increasing significantly, particularly in regions with high rates of hepatitis B and C viral infections, as well as in patients with chronic liver disease and cirrhosis [2, 3]. According to the Global Cancer Observatory, the estimated number of new HCC cases in 2020 is approximately 905,677, with an alarming mortality rate that highlights the urgent need for effective therapeutic strategies [4]. Despite progress in the management of HCC, therapeutic options remain scarce, especially for patients with advanced disease [5]. Liver transplantation (LT) is considered a curative treatment for early stage hepatocellular carcinoma (HCC). However, HCC recurrence remains a significant concern, with a recurrence rate of approximately 15% and a median time to recurrence of just over one year [6]. LT is performed in most patients who do not fulfill the Milan criteria, and recurrent HCC after LT is one of the most crucial issues to be addressed [7]. The high recurrence rates and associated poor prognosis underscore the urgent need for novel therapeutic strategies to improve outcomes for liver transplantation patients with HCC. Current systemic treatments, such as sorafenib and lenvatinib, have shown limited effectiveness, particularly in advanced stages of the disease [8]. Regarding the recurrence of liver cancer after liver transplantation, potential treatment options include curative intent, such as surgical resection or ablation, and palliative options, including intra-arterial therapies (chemo- and radioembolization) and/or systemic chemotherapy [9]. However, the outcomes are generally poor, with published literature indicating that the median time to HCC recurrence is 12.2–13 months after liver transplantation [10, 11]. Therefore, there is an urgent need for innovative treatment modalities that can precisely target recurrent HCC in liver transplantation patients and improve survival rates.

Natural killer (NK) cells are effector innate lymphoid cells that originate from bone marrow progenitor cells [12]. NK cells constitute a subset of lymphocytes characterized by the expression of CD56 and absence of T-cell receptor (CD3) expression [13]. NK cells can eliminate target cells without prior sensitization through inhibitory receptor recognition (the missing-self hypothesis) and activation of receptor recognition [14]. NK cells are renowned for their potent antitumor capabilities, which are achieved through direct cytotoxicity against tumor cells, cytokine production, and modulation of the immune microenvironment [15]. NK cells rapidly activate and mount cytotoxic attacks on stressed, aged, virus-infected, and cancerous cells, independent of prior antigen presentation via MHC I.

The quantity and functionality of NK cells are weakened in HCC patients [16], suggesting that the impaired function of NK cells may account for the failure of antitumor immune responses after LT in HCC patients. Given that the immunosuppressive regimen currently employed after organ transplantation reduces adaptive immune components while effectively preserving the innate components of cellular immunity [17], enhancing the NK cell response could be a promising immunotherapeutic strategy [18]. Clinical studies on the safety and efficacy of NK cells have been reported for leukemia [19, 20], lymphoid tumors [21], recurrent pediatric medulloblastoma and ependymoma [22], high-risk neuroblastoma in children [23], breast cancer [24], non-small cell lung cancer [25, 26], and renal cancer [27]. In the context of liver cancer patients who have undergone liver transplantation, Ohira et al. utilized NK cells derived from donor liver perfusate as a strategy to prevent HCC recurrence after LT [17]. Despite these promising findings, there are currently no published studies on the use of NK cells specifically to treat HCC recurrence after LT. This gap underscores the need for rigorous clinical investigations to evaluate the safety, feasibility, and efficacy of NK cell therapy in this unique patient population.

This Phase I dose-escalation clinical study aimed to explore the use of NK cells derived from the peripheral blood of relatives as a therapeutic approach for patients with recurrent HCC following liver transplantation. The primary objective was to assess the safety and tolerability of treatment. The secondary objective was to evaluate the efficacy of NK cells by measuring progression-free survival (PFS) and overall survival (OS). Through this study, we hope to provide valuable insights into the potential role of NK cells in managing recurrent liver cancer in a post-transplant setting, ultimately improving patient outcomes and expanding therapeutic options.

## Methods

### Study design and patient enrollment

This Phase 1 dose-escalation study was designed as a prospective, open-label, single-center trial to evaluate the safety and efficacy of NK cell therapy in patients with recurrent HBV-related HCC after liver transplantaon at the Third Affiliated Hospital of Sun Yat-sen University. The dose-escalation segment evaluated two NK cell dose levels: dose level one at 5 × 10^9^ cells per infusion and dose level two at 1 × 10^10^ cells per infusion. Eligible patients were allocated into four treatment groups based on a predefined dose-escalation and schedule-expansion scheme, rather than through randomization. The assignment aimed to explore different dose-frequency combinations and was conducted sequentially as patients were enrolled.The treatment regimens were as follows: Group A (low-dose NK cells, 4 infusions): patients received 5 × 10^9^ NK cells per infusion, which were administered every two weeks for a total of four infusions over two months. Group B (normal-dose NK cells, 4 infusions): Patients received 1 × 10^10^ NK cells per infusion, which were administered every two weeks for a total of four infusions over two months. Group C (normal-dose NK cells, 8 infusions): Patients received 1 × 10^10^ NK cells per infusion, which were administered every two weeks for a total of eight infusions over four months.

Group D (Incremental Dose NK Cells, 16 Infusions): Patients initially received 5 × 10^9^ NK cells per infusion every two weeks for the first two months (four infusions), followed by 1 × 10^10^ NK cells per infusion every two weeks for the next six months (​twelve​ infusions), totaling 16 infusions.

Written informed consent was obtained in accordance with the principles of the Declaration of Helsinki. This study was approved by the Ethics Committee of the Third Affiliated Hospital of Sun Yat-sen University (Approval No. (2014)2-126). This trial has been registered at ClinicalTrials.gov (NCT02399735). Patients were enrolled based on the following criteria: (a) adults aged 18–65 years; (b) had undergone liver transplantation for liver cancer and were pathologically confirmed to have hepatocellular carcinoma (HCC); (d) experienced recurrence detected by CT or MRI after liver transplantation; and (e) agreed to participate in the research program and signed the informed consent form. Patients were excluded if they (a) had autoimmune diseases requiring long-term glucocorticoid therapy, (b) were concurrently receiving any other investigational drug, (c) had an immunosuppressive or immunodeficient condition, including human immunodeficiency virus (HIV), (d) were pregnant or breastfeeding, or (e) were judged by investigators to be otherwise ineligible for the study.

### Definition of conventional treatment

Conventional treatment for patients with recurrent liver cancer after liver transplantation is a comprehensive approach that integrates multiple standard medical interventions, including concomitant anti-carcinoma and immunosuppressive therapies.

### NK cells manufacture

Peripheral blood (30–60 ml) was collected from patients’ healthy relatives by matching ABO blood types to isolate peripheral blood mononuclear cells (PBMCs). The isolated PBMCs were subsequently cultured in vitro for 14 days. Importantly, donor selection was not restricted based on HLA or KIR criteria, as our study aimed to evaluate the therapeutic potential of NK cells without the added complexity of donor-recipient compatibility optimization. This approach allowed us to streamline the donor recruitment process and focus on the innate immune mechanisms of NK cells, which have been shown to exert robust anti-tumor effects independently of HLA matching or KIR ligand mismatch.During the process of inducing NK cell proliferation, ALyS505NK-AC medium (Cell Science & Technology Institute Inc., Sendai, Japan) containing IL-2, IL-15, and IL-21 was added. The cells were cultured at 37 °C and 5% CO2 for seven days. The cell suspension from the culture flask was then transferred to a culture bag and ALyS505NK-EX medium (Cell Science & Technology Institute Inc., Sendai, Japan) containing IL-2 was added. The culture was maintained for days 12–14, and after successful expansion, samples were collected to detect cell phenotypes, fungi, bacteria, mycoplasma, endotoxins, and other indicators. ​For quality control, the release criteria for NK cell products were established as follows: cell viability > 90%, purity of CD56⁺CD3⁻ cells > 80%, sterility (confirmed by negative results of bacterial and fungal cultures), and endotoxin level < 5 EU/kg. All these criteria were strictly verified prior to the application of NK cell products to ensure their safety and functional validity.

### Outcomes

The primary objectives were to evaluate safety by documenting the frequency and severity of adverse events and identifying dose-limiting toxicities. To facilitate a thorough safety assessment, the participants underwent a comprehensive series of physical examinations and laboratory tests, including hematology, clinical chemistry, and urine analysis. Adverse events were systematically coded via Version 21.1 of the Medical Dictionary for Regulatory Activities (MedDRA) Terminology, and their severity was graded according to the NCI-CTCAE version 4 guidelines.

In addition to recording adverse events according to NCI-CTCAE version 4, we instituted comprehensive, transplant-specific safety monitoring to rigorously assess the impact of NK cell infusions. This included close surveillance for acute or chronic allograft rejection. Liver function tests, including alanine aminotransferase (ALT), aspartate aminotransferase (AST), total bilirubin, alkaline phosphatase (ALP), gamma-glutamyl transferase (GGT), albumin, and prothrombin time, were performed before each infusion and during follow-up visits. Viral reactivation was systematically monitored by quantifying serum DNA levels for hepatitis B virus (HBV) using polymerase chain reaction (PCR) at baseline, during the treatment period, and periodically during follow-up. Trough levels of immunosuppressive drugs (Tacrolimus and Sirolimus) were measured routinely to ensure they remained within the therapeutic range. Any dose adjustments to immunosuppressive regimens, whether due to toxicity, infection, or rejection, were meticulously documented throughout the study period. All such events, irrespective of their assessed causality to the NK cell infusion, were documented as treatment-emergent adverse events (TEAEs). We prospectively monitored for this predefined set of events of special interest (including liver function abnormalities, HBV reactivation, allograft rejection, and sepsis) to comprehensively capture the safety profile in this unique patient population.

For dose-limiting toxicity (DLT) assessment in this study, the definition was prespecified as follows: Grade ≥ 3 non-hematologic toxicity or Grade 4 hematologic toxicity that was confirmed to be associated with NK cell infusion, with such adverse events occurring within 7 days post-infusion.

The secondary endpoints included efficacy, pertaining to the effectiveness of NK cells, which was assessed through progression-free survival (PFS) in accordance with the RECIST 1.1 criteria, as well as overall survival (OS).

### Statistical analysis

Survival curves were constructed using the Kaplan–Meier method and compared between groups using log-rank tests. Survival curves were plotted using GraphPad Prism 5 (GraphPad Software, USA), taking advantage of their superior graphical capability. Multivariable Cox regression models were used to assess independent associations, adjusting for age and time to recurrence. Group A (the lowest-dose group) served as the reference category.All statistical analyses were performed using SPSS (version 18), owing to its comprehensive statistical analysis functions. Differences from these comparative tests were deemed significant if the P values were less than 0.05.

## Results

### Flow cytometry analysis of NK cells

Flow cytometric analysis of NK cells on day 14 after expansion revealed similar proportions of CD56⁺CD3⁻ NK cells across the four experimental groups. As shown in Supplementary Fig. 1A, the percentage of CD56⁺CD3⁻ NK cells was evaluated in 18 donors, with the data presented as scatter plots, indicating the median and range for each group. Groups A (*n* = 12), B (*n* = 20), C (*n* = 48), and D (*n* = 64) displayed comparable distributions of the NK cell subsets. As shown in Supplementary Fig. 1B, the level of T-cell contamination was consistently low in all groups and for all patients.

### Patient characteristics

**Fig. 1:**
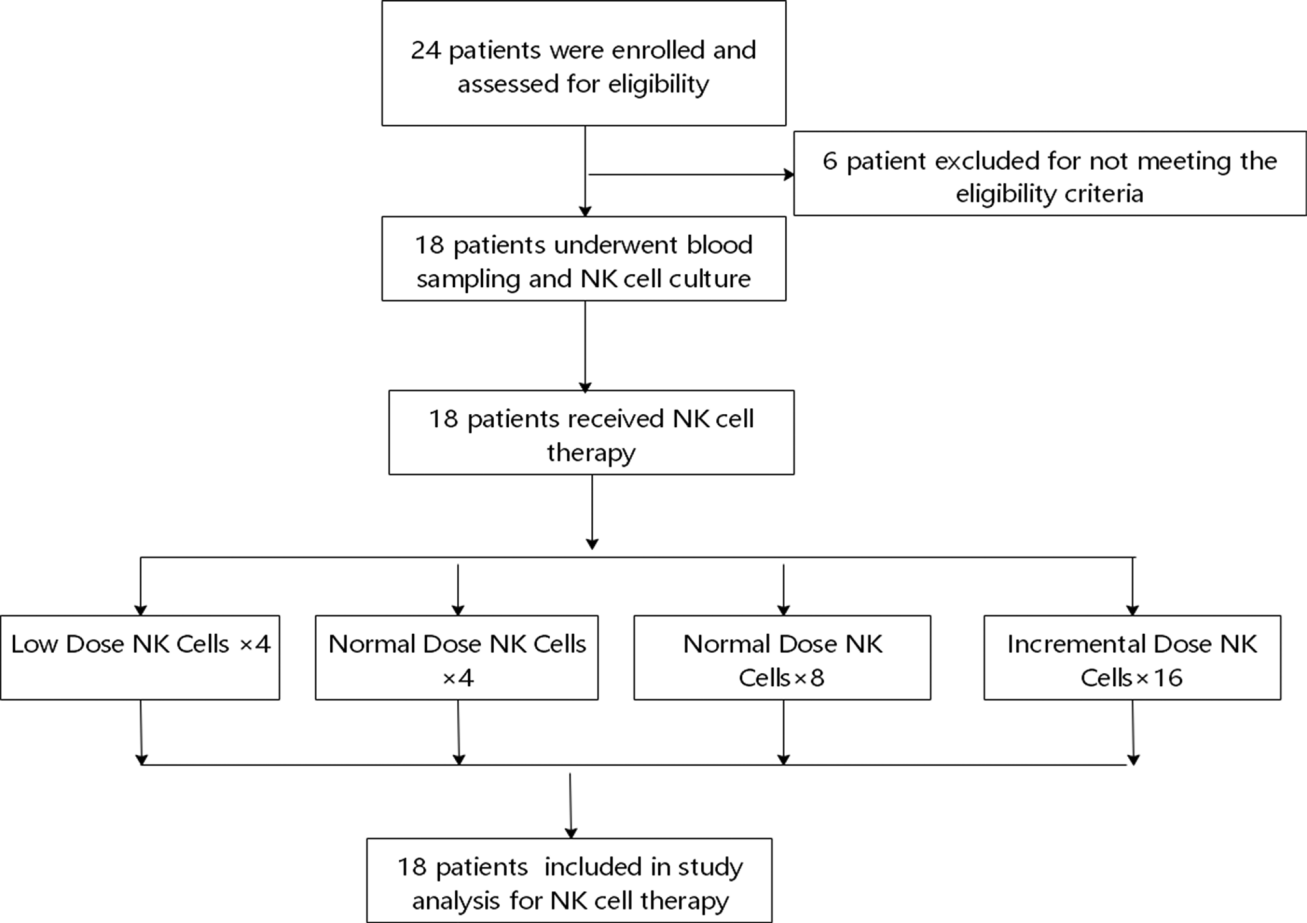
The enrolled patients were divided into four groups on the basis of cell dose and infusion schedule. Group A received conventional treatment along with NK cell infusions every two weeks for the first two months, totaling four infusions, at a dose of 5 × 10^9^ NK cells per infusion. Group B also received conventional treatment and NK cell infusions every two weeks for the first two months but at a higher dose of 1 × 10^10^ NK cells per infusion, totaling four infusions. Group C received conventional treatment with NK cell infusions every two weeks for the first four months, totaling eight infusions, at a dose of 1 × 10^10^ NK cells per infusion. Group D followed an incremental dosing schedule: initially receiving conventional treatment and low-dose NK cell infusions (5 × 10^9^ NK cells per infusion) every two weeks for the first two months (four infusions), then high-dose NK cell infusions (1 × 10^10^ NK cells per infusion) every two weeks for the subsequent six months (twelve infusions). *AE: adverse event*,* G: grade*,* NA: not applicable*,* NK: natural killer*

From December 31, 2014, to March 29, 2017, a total of 24 patients with recurrent HCC following liver transplantation were enrolled and assessed for eligibility. Six patients who did not meet these criteria were excluded from this study. The cutoff date for the analyses was September 30, 2024. Eighteen patients underwent blood sampling and NK cell culture and were randomly divided into four groups (Fig. 1). Group A (*n* = 3) received conventional treatment along with NK cell infusions every two weeks for the first two months, totaling four infusions, at a dose of 5 × 10^9^ NK cells per infusion. Group B (*n* = 5) also received conventional treatment and NK cell infusions every two weeks for the first two months, but at a higher dose of 1 × 10^10^ NK cells per infusion, totaling four infusions. Group C (*n* = 6) received conventional treatment with NK cell infusions every two weeks for the first four months, totaling eight infusions, at a dose of 1 × 10^10^ NK cells per infusion. Group D (*n* = 4) followed an incremental dosing schedule: initially receiving conventional treatment and low-dose NK cell infusions (5 × 10^9^ NK cells per infusion) every two weeks for the first two months (four infusions), followed by high-dose NK cell infusions (1 × 10^10^ NK cells per infusion) every two weeks for the subsequent six months (twelve infusions) (Table 1). Manufacturing failure did not occur during the production of NK cell products.

**Table 1:** Patient grouping by NK cell dosage and infusion frequency

Group	NK Cells Dose	Number of Infusions	Infusion Frequency	Duration of NK Cell Therapy	Number of enrolled patients
Group A	Low Dose (5 × 10^9^)	4	Every two weeks	Two months	3
Group B	Normal Dose (1 × 10^10^)	4	Every two weeks	Two months	5
Group C	Normal Dose(1 × 10^10^)	8	Every two weeks	Four months	6
Group D	Incremental Dose(5 × 10^9^,1 × 10^10^)	16(5 × 10^9^ cells, 4 times; 1 × 10^10^ cells,12 times)	Every two weeks	Eight months	4

The enrolled patients were divided into four groups based on the cell dose and infusion schedule

Group A (low-dose NK cells ×4): This group received conventional treatment along with NK cell infusions every two weeks for the first two months (total of 4 infusions) at a dose of 5 × 10^9^ NK cells per infusion

Group B: Normal-dose NK cells ×4: Patients in this group received conventional treatment and NK cell infusions every two weeks for the first two months (total of four infusions) at a dose of 1 × 10^10^ NK cells per infusion

Group C (Normal-dose NK cells×8): This group received conventional treatment and NK cell infusions every two weeks for the first four months (total of 8 infusions) at a dose of 1 × 10^10^ NK cells per infusion

Group D: Incremental Dose of NK Cells: Patients in this group received conventional treatment and NK cell infusions every two weeks according to the following schedule:

Low-dose NK cells ×4: A dose of 5 × 10^9^ NK cells per infusion was used for the first two months (total of four infusions)

Normal-dose NK cells ×12: A dose of 1 × 10^10^ NK cells per infusion was used

Conventional treatment” for patients with recurrent liver cancer after liver transplantation is a comprehensive approach that integrates multiple standard medical interventions, including concomitant anti-carcinoma and immunosuppressive therapies (Table 2).

**Table 2:** Baseline characteristics and concomitant therapies of patients receiving NK-cell treatment period

Patient ID	Group	Sex	Age(years)	ECOG performance status	BCLC stage	Milan criteria	Days of recurrence after liver transplantation	Location of Recurrent HCC	Prior treatment	Concomitant Anti-carcinoma Therapy	Concomitant Immunosuppressive Therapy	AFP (ng/ml)	Follow- up (months)
1	D	Male	44	1	C	Out	189	Liver	TACE, Sorafenib	TACE, TARE, RFA, Sorafenib, Apatinib	Tacrolimus, Sirolimus	2.44	22.9
2	D	Male	39	1	C	Out	145	Lung	None	Sorafenib, Xeloda	Tacrolimus, Sirolimus	17.07	15.4
3	D	Male	29	1	C	Out	150	Lung	None	Sorafenib, Radiotherapy, chemotherapy, TACE	Tacrolimus, Sirolimus, MMF	318.16	14.1
4	A	Male	65	1	C	Out	27	Liver, Bone, Chest wall, Lung	Chest wall tumor resection	Sorafenib, Xeloda, Chest wall tumor resection, Right lower lobectomy	Tacrolimus, Sirolimus	> 2000	10.8
5	A	Male	41	1	C	Out	39	Lung	None	Sorafenib	Tacrolimus, Sirolimus	2.06	12.3
6	A	Male	46	1	C	Out	147	Liver, Lung	Metastatic liver tumor resection	Sorafenib	Tacrolimus, Sirolimus	39.32	3.8
7	D	Male	49	1	C	Out	57	Liver	Radiofrequency ablation	None	Tacrolimus, Sirolimus, MMF	5.18	108.1
8	B	Male	55	1	C	Out	449	Liver	TACE	None	Tacrolimus, Sirolimus	25.45	14.3
9	B	Male	62	1	C	Out	88	Liver, lung	TACE	TACE, TARE, Apatinib	Tacrolimus, Sirolimus, MMF	1430.39	5.6
10	B	Male	35	1	C	Out	169	Liver, lung	None	TACE, Regorafenib	Tacrolimus, Sirolimus, MMF	18.28	17.4
11	B	Male	71	1	C	Out	87	Liver, Lung	Apatinib	Apatinib	Tacrolimus, Sirolimus	6818	3.0
12	B	Male	34	1	C	Out	188	Lung	Apatinib	Lenvatinib	Tacrolimus, Sirolimus, MMF	26.84	17.5
13	C	Male	45	1	C	Out	843	Lung	Targeted therapy, specifics not detailed	RFA, Sorafenib	Tacrolimus, Sirolimus, MMF	209.17	47.3
14	C	Male	50	1	C	Out	92	Liver, lung	Sorafenib	Apatinib	Tacrolimus, Sirolimus	4341.6	19.1
15	C	Male	48	1	C	Out	310	Liver, lung	TACE	TACE, Sorafenib	Tacrolimus, Sirolimus	10.6	20.5
16	C	Male	57	1	C	Out	99	Lung, Multiple metastases to the vena cava, Peritoneal metastases	None	None	Tacrolimus, Sirolimus	538.6	3.1
17	C	Male	32	1	C	Out	31	Lung	None	Apatinib	Tacrolimus, Sirolimus, MMF	102.69	8.9
18	C	Male	54	1	C	Out	117	Abdominal cavity, Lung	Sorafenib	Sorafenib	Tacrolimus, Sirolimus	314.1	3.3

NK: natural killer, TACE: transcatheter arterial chemoembolization, RFA: radiofrequency ablation, TARE: transarterial radioembolization. All concomitant anti - carcinoma and immunosuppressive therapies listed were administered during the NK cell infusion period

Concomitant Anti-carcinoma Therapy encompasses various treatments, such as surgical interventions, including tumor resection (including removal of metastatic tumors in the liver and chest wall when clinically indicated) and lobectomy. Interventional therapies, such as transarterial chemoembolization (TACE), are integral components of conventional treatment regimens. Drug-based therapies, including targeted therapies (e.g., Sorafenib, Apatinib) and chemotherapy, are used for effective management. Radiation therapy is used for local tumor control, and physical ablation techniques such as radiofrequency ablation (RFA) are considered. The selection of these treatments is tailored to each patient’s unique condition, considering factors such as tumor stage, metastasis status, and overall health. Patients received a complex array of concomitant treatments alongside NK cell infusions. We performed statistical analysis of concomitant therapies distribution across groups using Fisher’s exact test. The results showed no significant differences in TACE (*P* = 0.65), RFA (*P* = 0.50), apatinib (*P* = 0.70) or surgical intervention (*P* = 0.17) distribution, suggesting balanced baseline characteristics. Although sorafenib use differed significantly among groups (*P* = 0.02), the cohorts with the best survival outcomes (Group C, 33.3%; Group D, 75%) did not correspond to those with the highest administration rates. This indicates that the survival benefit cannot be attributed solely to sorafenib exposure(Supplementary Table 2)This timeline outlines the temporal relationships and overlaps between NK cell therapy, systemic therapies, locoregional interventions, and immunosuppressive regimens༈Supplementary Fig. 2༉. Concomitant Immunosuppressive Therapy includes the use of drugs, such as Tacrolimus, Sirolimus, and Mycophenolate Mofetil (MMF).

The baseline characteristics of the patients included in the study are summarized in Table 2. The median follow-up was 14.2 months (range: 3.0–108.1 months). The cohort consisted predominantly of male patients (*n* = 18), with a median age of 47 years (range: 29–71 years). All patients were classified as having an Eastern Cooperative Oncology Group (ECOG) performance status of 1 and were at BCLC stage C. All patients exceeded the Milan criteria, as determined by pathological findings. The median time to recurrence after liver transplantation was 145 days, with the liver and lungs being the most common sites of recurrence. Prior treatments varied, with some patients receiving TACE, sorafenib, or radiofrequency ablation. Concomitant anti-carcinoma therapies included sorafenib, apatinib, and TACE, whereas immunosuppressive therapy was consistently maintained with tacrolimus and sirolimus. The descriptive statistics for the patients enrolled in each group are presented in Supplementary Table 1. Age in group A was 50.67 ± 12.17 years, in group B was 51.40 ± 12.43 years, in group C was 48.50 ± 9.17 years, and in group D was 40.25 ± 8.50 years.

In terms of the median number of days of recurrence after liver transplantation, Group A had a median of 39 days (range: 27–147 days), Group B had 169 days (range: 87–449 days), Group C had 108 days (range: 31–843 days), and Group D had 147.5 days (range: 57–189 days). Regarding the median follow - up time, group A had 10.8 months (range: 3.8–12.3 months), group B had 14.3 months (range: 3.0–17.5 months), group C had 14.0 months (range: 3.1–47.3 months), and group D had 19.15 months (range: 14.1–108.1 months).

### Treatment-related adverse events

The treatment-related adverse events (AEs) observed in patients receiving NK cell infusions are detailed in Table 3. Eighteen patients received varying numbers of infusions, with treatment durations ranging from 56 days to 224 days. The most common AE was Grade 1 pyrexia, observed in multiple patients at different infusion numbers and dose levels. Notably, Grade 2 pyrexia has been reported in a few cases. The onset of AEs typically occurs shortly after infusion, with most AEs resolving within a day. As shown in Table 4, the most frequently reported AE was pyrexia, which occurred in 7 out of 18 participants (38.9%), Notably, no instances of Grade 3 or Grade 4 pyrexia were observed. Importantly, other monitored AEs—including alanine aminotransferase increase, hepatitis B virus DNA increase, hyperbilirubinemia, acute graft rejection, chronic graft rejection, graft dysfunction, and sepsis—were entirely absent across all grades (Grades 1–4) in this cohort. Collectively, the data presented in Tables 3 and 4 provide a comprehensive overview of the treatment-emergent safety profile. The spectrum of adverse events directly associated with NK cell infusion was narrow, primarily consisting of transient, low-grade pyrexia. More importantly, prospective monitoring for pre-specified events of special interest in this transplant population—including liver injury, viral reactivation, allograft rejection, and severe infection—confirmed the absence of such complications. This favorable safety observation, occurring alongside stable immunosuppressive drug levels (Supplementary Fig. 3), strongly supports the tolerability of allogeneic NK cell therapy in liver transplant recipients.

**Table 3:** Treatment-Related adverse events

PatientIdentifer	Number of infusions received	Duration of treatment (days)	AE probably or possibly related to NK cells			
			AE	Duration of AE (days)	Time to AE onset post infusion (days)	Associated infusion number & dose level
1	16	224	G1 pyrexia	1	0	7th infusion at 1 × 10^10^ cells
			G2 pyrexia	1	0	10th infusion at 1 × 10^10^ cells
2	16	224	G1 pyrexia	1	0	10th infusion at 1 × 10^10^ cells
3	16	224	G1 pyrexia	1	0	5th infusion at 1 × 10^10^ cells
			G1 pyrexia	1	0	12th infusion at 1 × 10^10^ cells
			G1 pyrexia	1	0	14th infusion at 1 × 10^10^ cells
			G1 pyrexia	1	0	15th infusion at 1 × 10^10^ cells
			G1 pyrexia	1	0	16th infusion at 1 × 10^10^ cells
4	4	56	None	NA	NA	NA
5	4	56	G1 pyrexia	1	0	2th infusion at 5 × 10^9^ cells
			G1 pyrexia	1	0	4th infusion at 5 × 10^9^ cells
6	4	56	G1 pyrexia	1	0	1th infusion at 5 × 10^9^ cells
			G1 pyrexia	1	0	2th infusion at 5 × 10^9^ cells
			G1 pyrexia	1	0	3th infusion at 5 × 10^9^ cells
7	16	224	None	NA	NA	NA
8	4	56	None	NA	NA	NA
9	4	56	None	NA	NA	NA
10	4	56	None	NA	NA	NA
11	4	56	None	NA	NA	NA
12	4	56	None	NA	NA	NA
13	8	112	None	NA	NA	NA
14	8	112	None	NA	NA	NA
15	8	112	G1 pyrexia	1	0	1th infusion at 1 × 10^10^ cells
			G1 pyrexia	1	0	2th infusion at 1 × 10^10^ cells
			G1 pyrexia	1	0	3th infusion at 1 × 10^10^ cells
16	8	112	None	NA	NA	NA
17	8	112	None	NA	NA	NA
18	8	112	G1 pyrexia	1	0	6th infusion at 1 × 10^10^ cells

**Table 4:** AEs related to NK cell infusion in participants

Preferred term, *n* (%)	Any grade (*n* = 18)	Grade 3 (*n* = 18)	Grade 4 (*n* = 18)
Pyrexia	7	0	0
Alanine aminotransferase increased	0	0	0
Hepatitis B virus DNA increased	0	0	0
Hyperbilirubinemia	0	0	0
Acute graft rejection	0	0	0
Chronic graft rejection	0	0	0
Graft dysfunction	0	0	0
Sepsis	0	0	0

This absence of severe or organ-specific toxicities suggests that NK cell infusion was well-tolerated without evidence of significant hepatotoxicity, rejection, or infectious complications under the study conditions. These findings support the feasibility of NK cell-based immunotherapy in this patient population, highlighting its potential for further clinical development with careful monitoring for febrile reactions as the primary manageable side effect. Therapeutic drug monitoring of tacrolimus and sirolimus revealed that trough concentrations remained stable within the expected therapeutic ranges throughout the study period, with no clinically significant fluctuations observed after NK cell infusion(Supplementary Fig. 3). The consistency of drug levels confirms that no dose modifications were necessary following NK cell infusion.This stability aligned with the favorable safety profile shown in Table 4, where no episodes of graft rejection or related adverse events were reported.

### Clinical efficacy

The survival outcomes of patients receiving NK cell infusions were evaluated using Kaplan-Meier analysis, which focused on both progression-free survival (PFS) and overall survival (OS) among four groups: Group A (four times at a low dose), Group B (four times at a normal dose), Group C (eight times at a normal dose), and Group D (16 times at an incremental dose).

### Progression-free survival (PFS)

**Fig. 2:**
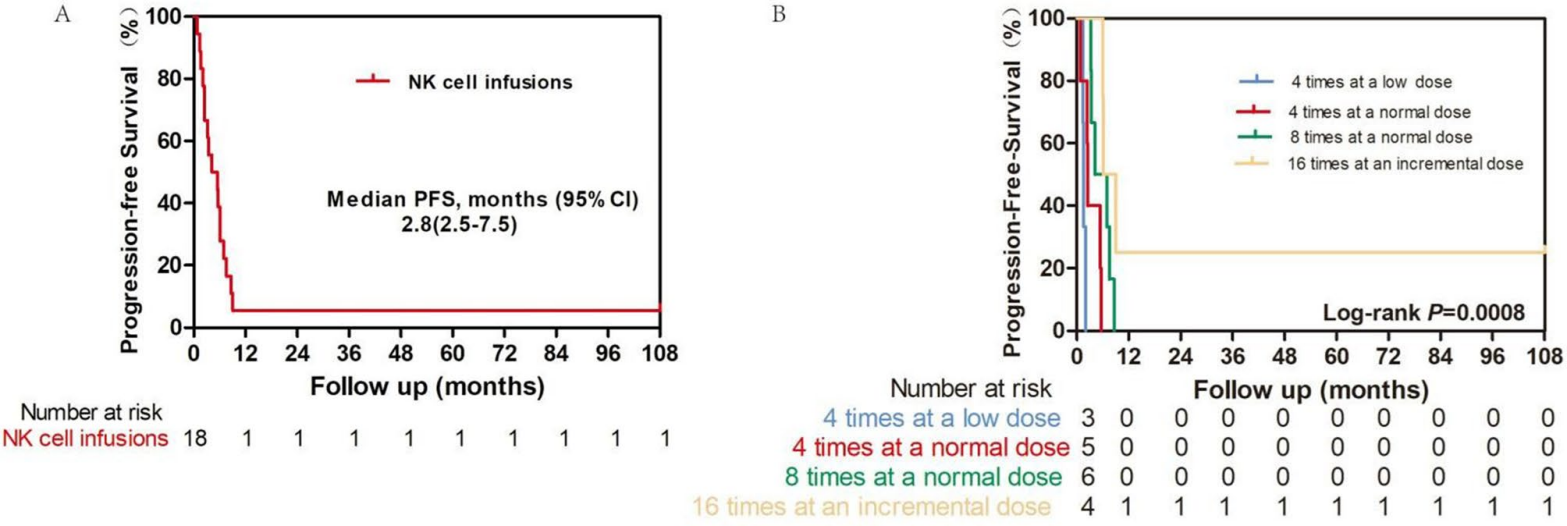
Kaplan-Meier estimates of Progression-free survival (PFS).(**A**) All patients. (**B**) Patients stratified by natural killer (NK) cell infusion groups

As depicted in Fig. 2A, the PFS curve for all patients showed a gradual decline in survival probability over time, with a median PFS of 4.8 months (CI: 2.5–7.5 months) for the cohort of 18 patients. Kaplan-Meier curves demonstrated significant differences in PFS probabilities among the treatment groups (log-rank *P* = 0.0008). The PFS was 1.6 months for Group A, 2.5 months for Group B, 5.5 months for Group C, and 7.5 months for Group D (Fig. 2B). Figure 2B further stratifies PFS based on the frequency and dosage of NK cell infusions. Patients who received infusions four times at a low dose had the lowest survival probability, whereas those who received infusions four times at a normal dose had improved outcomes. Notably, patients who were treated for 16 cycles at an incremental dose ( Group D) demonstrated the highest survival probability, suggesting a dose-response relationship. The log-rank test yielded a P value of less than 0.05, indicating statistically significant differences among the dosing groups.

### Overall survival (OS)

**Fig. 3:**
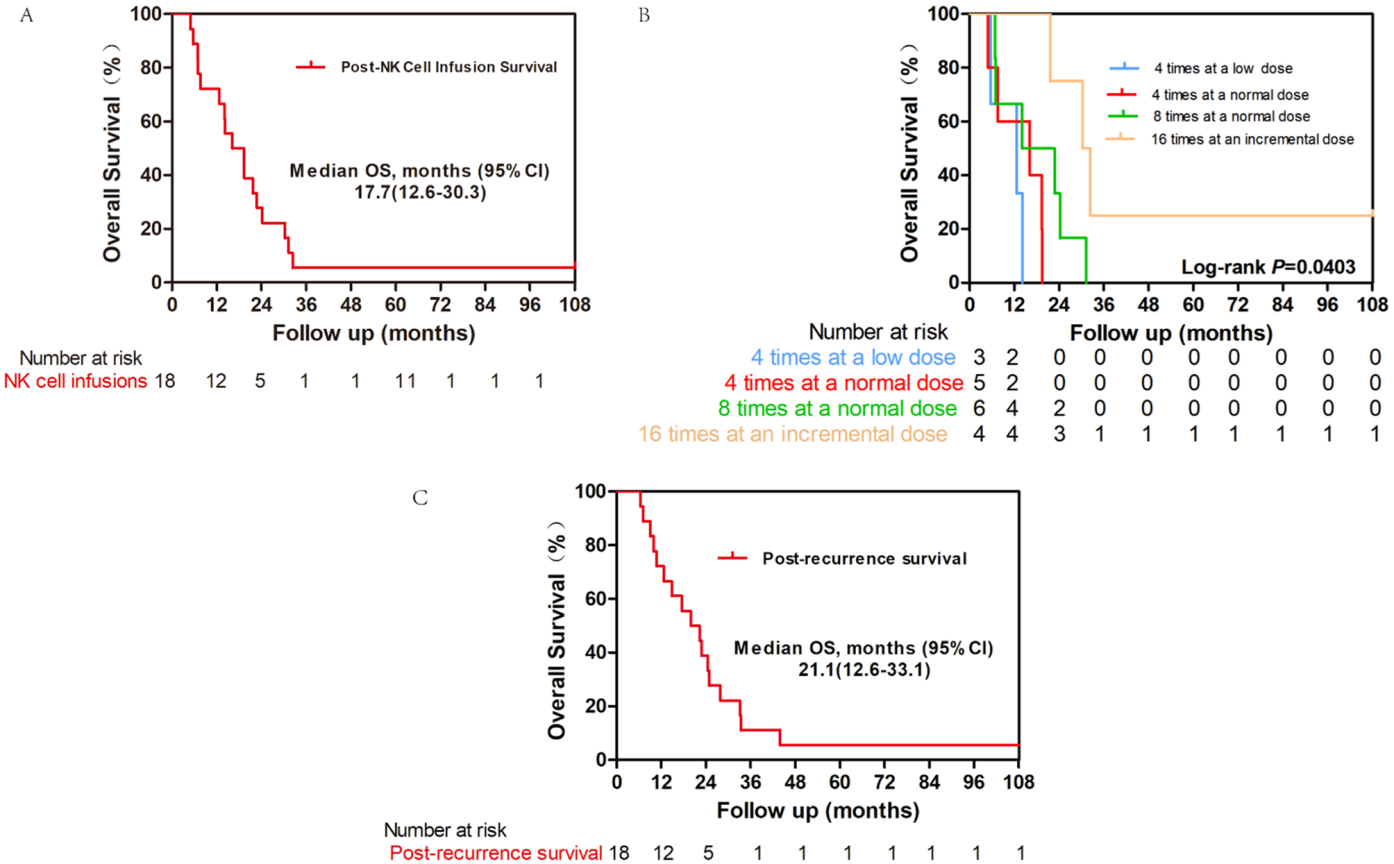
Kaplan-Meier estimates of overall survival (OS).​​ (**A**) OS of all patients. (**B**) OS stratified by natural killer (NK) cell infusion groups. (**C**) OS following recurrence (median OS 21.1 months, 95% CI: 12.6–33.1 months)

Figure 3A shows the overall survival (OS) curve for all patients, revealing a sharp decline in survival probability within the first 12 months. The median OS for the cohort of 18 patients was notably short, at 17.7 months (CI: 12.6–30.3 months). Kaplan-Meier curves demonstrated significant differences in OS probabilities among the treatment groups (log-rank *P* = 0.0403). The OS was 12.6 months for Group A, 16.1 months for Group B, 18.4 months for Group C, and 31.3 months for Group D (Fig. 3B). Figure 3B provides a stratified analysis of OS based on the frequency and dosage of NK cell infusion. Patients receiving four infusions at a low dose had the poorest survival outcomes, whereas those receiving four infusions at a normal dose showed slight improvement. Patients treated four times at incremental doses presented the best survival outcomes, highlighting the potential benefit of increasing the dosage. The log-rank test produced a p-value of less than 0.05, confirming significant differences in OS among the dosing groups. On September 31, 2024, patient 7 survived and underwent curative radiofrequency ablation (RFA). Since RFA, the patient has been undergoing regular follow-up examinations, and to date, no recurrence has been detected.

Collectively, these findings suggest that both the frequency and dosage of NK cell infusions are critical factors that influence survival outcomes, underscoring the importance of optimizing treatment regimens to increase both PFS and OS in patients.

### Correlation analysis

We performed Spearman correlation tests across the entire cohort (*n* = 18) between the continuous baseline variables “Age” and “Days to Recurrence” and the survival endpoints PFS and OS. The results showed no statistically significant correlations (all *p* > 0.05) in this small data set(Supplementary Table 3).

In additional, we performed an exploratory Spearman correlation analysis to assess whether the purity of the infused NK cell products (percentage of CD56⁺CD3⁻ cells) was associated with patient survival. The analysis, which included data from all infusion batches, found no statistically significant correlation between product purity and either Progression-Free Survival (PFS) or Overall Survival (OS) (Spearman’s ρ for PFS: [ρ value= -0.131, *P* = 0.604]; for OS: [ρ value= -0.069, *P* = 0.785]). These results are now presented in Supplementary Table 4. While this analysis in our small cohort does not prove an absence of effect, it provides no evidence that the observed variation in purity (within the > 80% release threshold) was a major driver of the differences in clinical outcomes.

To further investigate the association between treatment regimens and survival while accounting for baseline variables, an exploratory multivariable Cox proportional hazards regression was performed. After adjusting for age and time to recurrence, treatment group remained a significant independent predictor of overall survival (omnibus *p* = 0.044). Compared to the reference Group A, Group D (incremental high-dose regimen) was associated with a significantly reduced hazard of death (Hazard Ratio [HR] = 0.027, 95% Confidence Interval [CI]: 0.002–0.382, *p* = 0.008). The hazard ratios for Groups B and C were not statistically significant (Supplementary Table 5). The effects of the covariates age (HR = 1.024, *p* = 0.419) and time to recurrence (HR = 0.997, *p* = 0.056) were not significant in this model. The confidence intervals for all estimates were wide, reflecting the limited sample size.

## Discussion

The adoptive transfer of ex vivo-expanded, relative-derived allogeneic NK cells was well-tolerated in this Phase I dose-escalation study, which is the first to evaluate this therapeutic approach for recurrent HCC following liver transplantation. Our data indicate a notable dose-response relationship, with a higher frequency and dosage of NK cell infusions being associated with increased PFS and OS.

All patients exceeded the Milan criteria, indicating advanced disease stages and a higher risk of post-transplantation recurrence [28]. In such patients, secondary liver transplantation and resection are not viable options after recurrence. Despite these traditional treatments, the survival rate for patients remains low, with a median overall survival (OS) of only 12.2–13 months [10, 11]. By identifying effective treatment options, we aimed to provide better management strategies for patients with recurrence after liver transplantation, ultimately leading to improved survival and quality of life. Consequently, NK cell infusions are being explored in clinical studies of patients with recurrent liver cancer post-transplantation to assess their potential to improve survival.

NK cell infusions were generally well tolerated, with grade 1 pyrexia being the most common adverse event. Seven patients (patients 1, 2, 3, 5, 6, 7, 15, and 18) experienced treatment-related adverse events, all of which were classified as grade 1 pyrexia. This finding is consistent with those of previous reports, where fever was commonly observed following NK cell infusions, likely due to cytokine release [29]. Similar to the findings by Yang et al. in a study of HBV-TCR-T cells in patients with recurrent hepatocellular carcinoma after liver transplantation, grade 1 pyrexia was also a common adverse event [30]. The fleeting nature of these adverse events emphasizes the safety profile of NK cell-based therapy, which is of paramount importance for its application in clinical scenarios. Notably, NK cells are derived from the peripheral blood of patients’ relatives and these NK cells are classified as allogeneic haploidentical NK cells. No severe adverse reactions were reported.

In our study, we did not observe any clinical or laboratory evidence of an anti-donor immune response after multiple infusions of allogeneic NK cells. This aligns with previous studies demonstrating that NK cells, as part of the innate immune system, are less likely to elicit stronger alloimmune reactions than T cells. The unique properties of NK cells, including their lack of T-cell receptor (TCR) expression and reliance on innate immune recognition mechanisms, contribute to their reduced immunogenicity [31]. Additionally, we carefully monitored patients for signs of graft-versus-host disease (GVHD) or other immune-mediated adverse events. Throughout the study period, no GVHD or other significant immune-mediated complications were observed. This is consistent with the well-documented safety profile of NK cell therapies, particularly haploidentical transplantations. The absence of graft rejection, dysfunction, or viral reactivation in our cohort, coupled with stable immunosuppressive drug levels, suggests that NK cell infusion is well-tolerated in liver transplant recipients. This aligns with emerging evidence on the safety of adoptive immunotherapy in immunocompromised populations.

Our Kaplan-Meier analysis revealed a median PFS of 4.8 months for the entire cohort. Stratified analysis based on the frequency and dosage of NK cell infusions revealed a dose-response relationship, indicating that patients receiving higher doses and more frequent infusions exhibited improved PFS. This finding is supported by previous studies, indicating that the antitumor activity of NK cells can be enhanced by increasing the dosage. The unique mechanisms of action of immune checkpoint inhibitors (ICIs) provide a critical context for interpreting our results. ICIs activate and reinvigorate the body’s immune response against tumors, which can introduce the possibility of a delayed treatment effect (DTE) [32]. This concept implies that therapeutic benefits may continue to emerge even after the initial signs of tumor progression. This delayed effect is particularly relevant in the context of NK cell therapy, as NK cells can exert significant immunomodulatory effects in the tumor microenvironment, potentially providing therapeutic benefits even when tumors progress.In alignment with this rationale, our protocol allowed patients to continue receiving NK cell infusions even after tumor progression. This approach was designed to maximize the potential benefits of NK cell therapy by leveraging the immunomodulatory effects of NK cells. By continuing NK cell infusions post-progression, we aimed to enhance overall treatment efficacy and improve patient outcomes.

Similarly, OS analysis revealed a median survival duration of 17.7 months, which is significantly longer than the typical 12.2–13 months observed in patients with recurrent HCC following LT [10, 11]. Importantly, survival duration is calculated from the initiation of NK cell therapy, and many patients begin treatment after experiencing recurrence. Furthermore, we found a median OS of 21.1 months (95% CI: 12.6–33.1 months) after recurrence (Fig. 3C), which is notably longer than the 12.2–13 months reported in previous studies of patients with recurrent HCC following LT.

The incremental dosing schedule in Group D, commencing with a low dose and escalating to a high-dose infusion, yielded the most favorable survival results. This implies that an incremental dosing strategy might assist in alleviating potential adverse effects, while still achieving therapeutic effectiveness. These outcomes, while modest, are significant considering the advanced stage of the disease (BCLC stage C) and poor prognosis typically associated with HCC exceeding the Milan criteria. The improved survival outcomes in patients receiving higher and more frequent doses of NK cells suggests that optimizing the dosing schedule could be a critical factor in treatment planning. The incremental dosing schedule in Group D may have allowed for gradual immune engagement, reducing early cytokine-related toxicity while promoting sustained NK cell activity and adaptive immune priming.

Our study adds to the evolving landscape of NK cell-based immunotherapy, a field with promising outcomes in hematologic malignancies. Notably, allogeneic NK cell infusions have demonstrated encouraging results in achieving remissions in acute myeloid leukemia (AML), often with a favorable safety profile and minimal induction of GVHD [33, 34]. However, the efficacy of NK cell therapy in solid tumors, including HCC, has been more challenging to establish, primarily due to the immunosuppressive tumor microenvironment and obstacles to effective tumor infiltration [35].Within the specific context of liver transplantation, strategies utilizing donor-derived cells have also been explored. For instance, Masahiro Ohira et al. investigated the adoptive transfer of NK cells isolated from donor liver perfusate, reporting a potential benefit in preventing HCC recurrence post-transplant [17]. While our approach differs—employing ex vivo expanded NK cells from related donors—it shares the foundational principle of harnessing innate immunity against recurrent malignancy. Our findings of a manageable safety profile, absence of GVHD or allograft rejection, align with the general consensus on the safety of allogeneic NK cell products in the post-transplant setting [36].

### Strengths and limitations

One of the advantages of this study is its innovation, as it is the first article reporting on NK cell therapy for patients with recurrent HCC after LT. Another advantage is the nine-year follow-up of patients, which provides a more accurate assessment of the adverse reactions and benefits of NK cell treatment following recurrence after transplantation. Moremover, NK cells are less likely to cause severe GVHD or rejection is a key strength [37].While the findings are encouraging, the limitations of this study, including its small sample size and lack of a control group that received conventional therapy alone, must be acknowledged. Second, the study employed a non-randomized design with sequential enrollment into different dose-frequency regimens, rather than a traditional dose-escalation schema. This approach, while enabling the exploration of multiple strategies, may introduce temporal or selection biases that cannot be fully adjusted for in the analysis. Third, the non-randomized allocation resulted in baseline imbalances between groups. Although post-hoc correlation analyses in our small cohort did not show significant associations between these specific variables (age, time to recurrence) and survival outcomes, their potential confounding influence cannot be entirely excluded. The small, non-randomized sample size and the heterogeneity of concomitant anticancer treatments received by patients are considerable limitations that preclude definitive conclusions on clinical efficacy. Although our post hoc analysis indicated no significant differences in the distribution of major concomitant therapies across groups, we cannot entirely rule out their potential influence as confounding factors. These factors limit the generalizability of the results and necessitate further validation through larger multicenter trials with robust control conditions. In the subsequent phase of our research, we will incorporate a larger sample size and implement longer-term follow-up assessments to monitor the occurrence of side effects. Fourth, NK cells were derived from different related donors without HLA/KIR matching. While all products met predefined release criteria, we did not perform standardized functional potency assays across all batches. Therefore, potential donor-to-donor variability in NK cell functional activity remains an unmeasured confounder that could have influenced outcomes. Fifth, one potential limitation pertains to the characteristics of the NK cell products themselves. Although all products met the predefined release criteria (viability > 90%, CD56⁺CD3⁻ purity > 80%), batch-to-batch variability in CD56⁺CD3⁻ purity was observed. To assess whether this variability could have driven the clinical outcomes, we performed an exploratory correlation analysis, which did not reveal a significant association between product purity and either PFS or OS in this cohort. Furthermore, the level of CD3⁺ T-cell contamination was minimal and consistent across all batches, which aligns with the favorable safety profile (absence of GVHD or rejection) observed in our study. Nevertheless, without standardized functional potency assays across all donors, we cannot definitively exclude donor-to-donor variability in NK cell functionality as an unmeasured confounder. Sixth, this study lacks pharmacokinetic and pharmacodynamic data on the infused NK cells, such as their in vivo persistence, expansion kinetics, or functional biomarkers. Consequently, we cannot elucidate the mechanistic basis for the observed clinical outcomes. Incorporating such translational endpoints in future studies is crucial to understanding the biology of NK cell therapy in this setting.

## Conclusions

Our exploratory study highlights that the infusions were predominantly safe and well-tolerated, preliminarily emphasizing the potential of tailored NK cell infusion strategies to influence survival outcomes in patients with recurrent HCC following LT. The observed dose-response relationship suggests that optimizing the frequency and dosage of NK cell infusions merits further investigation to enhancing therapeutic efficacy. Therefore, they should be considered hypothesis-generating. Future multicenter randomized controlled trials should be designed to further investigate the effectiveness of NK cell therapy in patients with recurrent HCC after liver transplantation.

## Supplementary Information

Below is the link to the electronic supplementary material.

Supplementary Material 1

Supplementary Material 2

Supplementary Material 3

Supplementary Material 4

## Data Availability

Supporting data for this study can be obtained from the corresponding authors and first authors upon reasonable request.

## Abbreviations

HCC: Hepatocellular carcinoma
NK: Natural killer
PFS: Progression-free survival
OS: Overall survival
LT: Liver transplantation
ADCC: Antibody-dependent cell-mediated cytotoxicity
PBMCs: Peripheral blood mononuclear cells
CT: Computed tomography
MRI: Magnetic resonance imaging
MedDRA: Medical Dictionary for Regulatory Activities
RECIST: Response Evaluation Criteria In Solid Tumors
CR: Complete response
PR: Partial response
GVHD: Graft-versus-host disease
TACE: Transarterial chemoembolization
RFA: Radiofrequency ablation
MMF: Mycophenolate Mofetil
ECOG: Eastern Cooperative Oncology Group
BCLC: Barcelona Clinic Liver Cancer
ICIs: Immune checkpoint inhibitors
DTE: Delayed treatment effect
