# Supplementary material for: NK cell infusion is well-tolerated and shows preliminary efficacy in patients with recurrent hepatocellular carcinoma post-liver transplantation : a phase I trial
Source: J Transl Med. 2026 Jan 24;24:261. doi: 10.1186/s12967-026-07725-x (PMC12911381; doi:10.1186/s12967-026-07725-x)
Supplement: Supplementary file 1 — Supplementary Material 1 [file 12967_2026_7725_MOESM1_ESM.pdf]

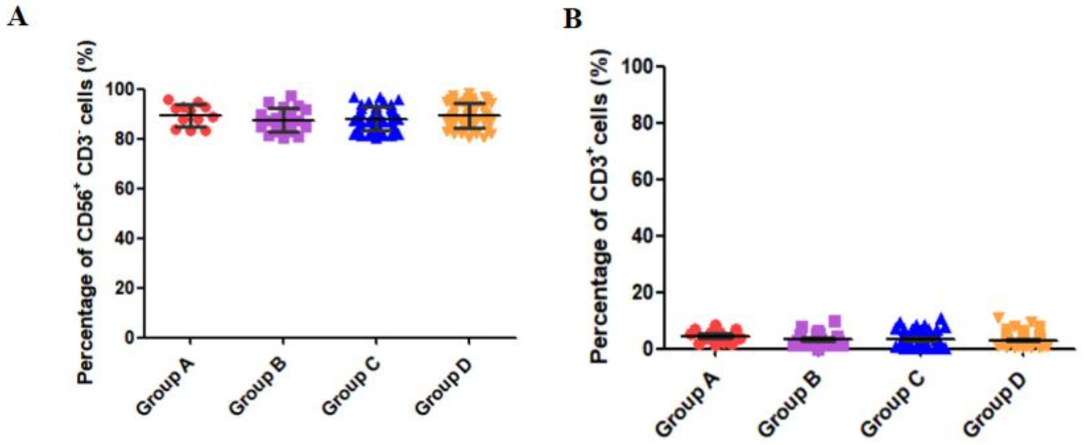

Supplementary figure 1. Phenotypic characterization of expanded cells from different groups. (A) Proportion of CD56⁺CD3⁻ (NK) cells. (B)Proportion of CD3⁺(T) cells. Data are presented as scatter plots showing the percentage for each donor, with the horizontal line representing the median. Group A: n = 12, Group B: n = 20, Group C: n = 48, Group D: n = 64.

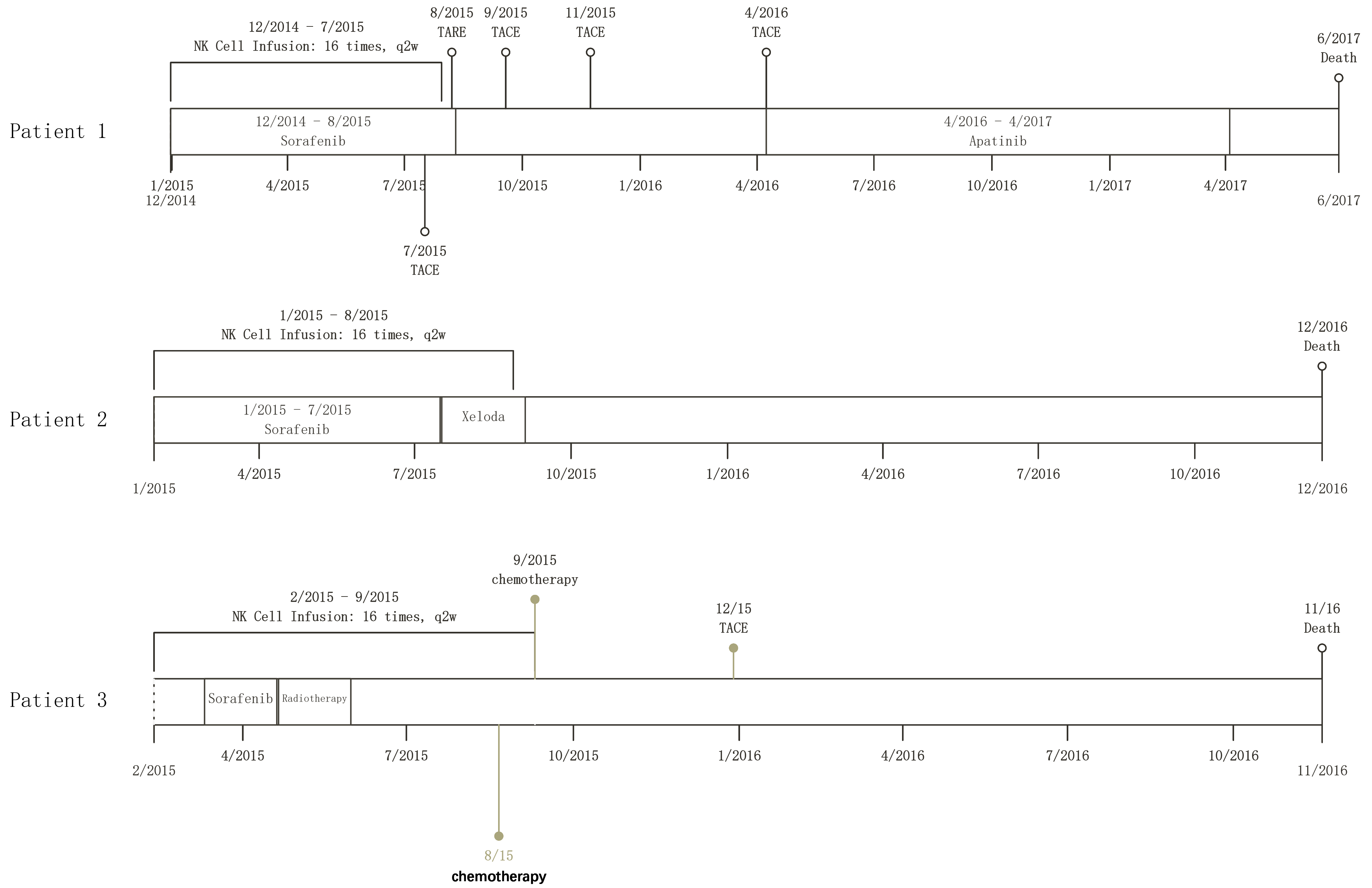
Patient 1
12/2014 – 7/2015
NK Cell Infusion: 16 times, q2w
8/2015
TARE
9/2015
TACE
11/2015
TACE
4/2016
TACE
6/2017
Death
12/2014 – 8/2015
Sorafenib
4/2016 – 4/2017
Apatinib
1/2015
12/2014
4/2015
7/2015
10/2015
1/2016
4/2016
7/2016
10/2016
1/2017
4/2017
6/2017
7/2015
TACE
Patient 2
1/2015 – 8/2015
NK Cell Infusion: 16 times, q2w
12/2016
Death
1/2015 – 7/2015
Sorafenib
Xeloda
1/2015
4/2015
7/2015
10/2015
1/2016
4/2016
7/2016
10/2016
12/2016
Patient 3
9/2015
chemotherapy
2/2015 – 9/2015
NK Cell Infusion: 16 times, q2w
12/15
TACE
11/16
Death
Sorafenib
Radiotherapy
2/2015
4/2015
7/2015
10/2015
1/2016
4/2016
7/2016
10/2016
11/2016
8/15
chemotherapy

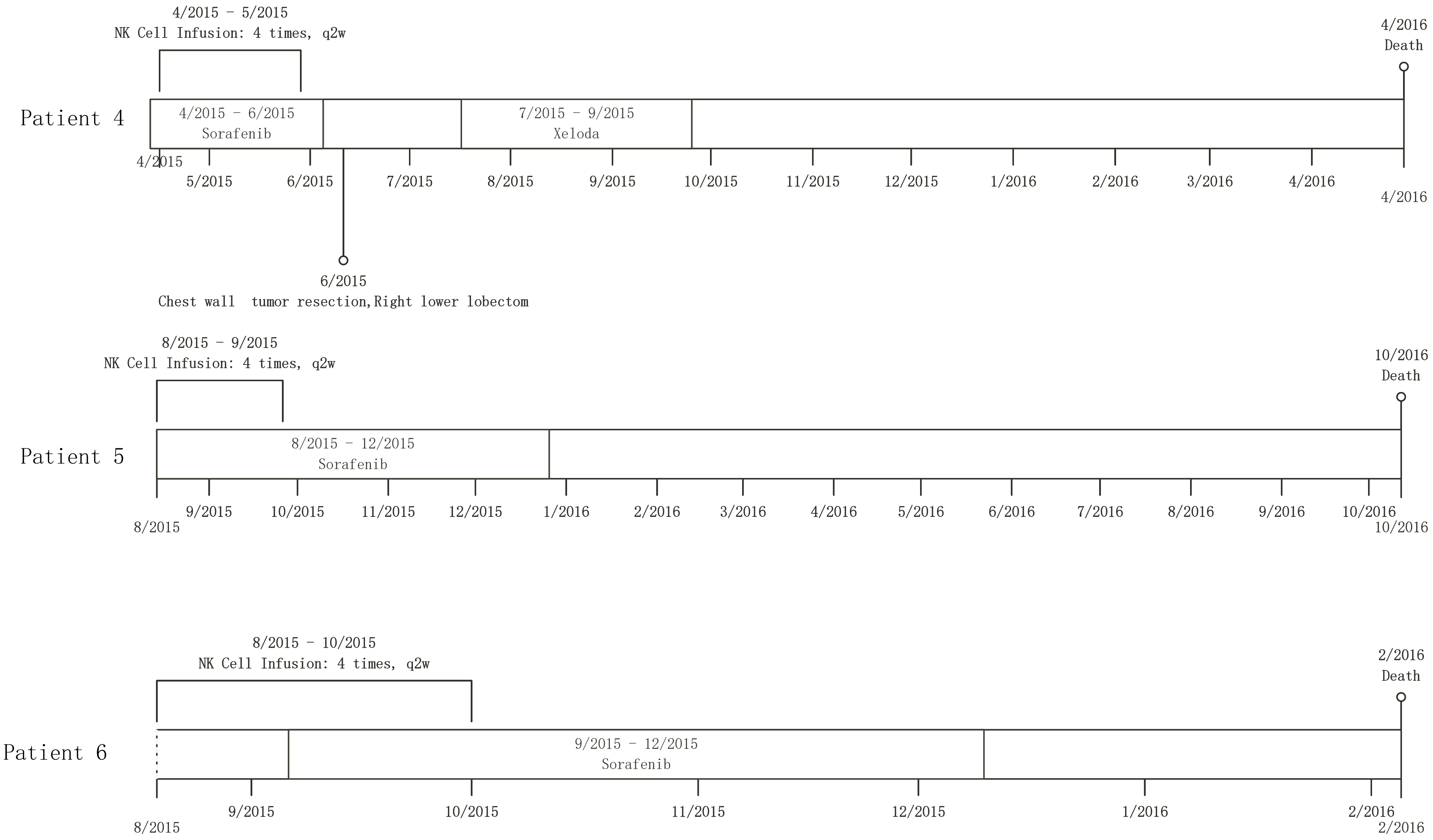

4/2015 - 5/2015
NK Cell Infusion: 4 times, q2w
4/2016
Death
Patient 4
4/2015 - 6/2015
Sorafenib
7/2015 - 9/2015
Xeloda
4/2015
5/2015
6/2015
7/2015
8/2015
9/2015
10/2015
11/2015
12/2015
1/2016
2/2016
3/2016
4/2016
4/2016
6/2015
Chest wall tumor resection,Right lower lobectom
8/2015 - 9/2015
NK Cell Infusion: 4 times, q2w
10/2016
Death
Patient 5
8/2015 - 12/2015
Sorafenib
9/2015
10/2015
11/2015
12/2015
1/2016
2/2016
3/2016
4/2016
5/2016
6/2016
7/2016
8/2016
9/2016
10/2016
8/2015
10/2016
8/2015 - 10/2015
NK Cell Infusion: 4 times, q2w
2/2016
Death
Patient 6
9/2015 - 12/2015
Sorafenib
9/2015
10/2015
11/2015
12/2015
1/2016
2/2016
8/2015
2/2016

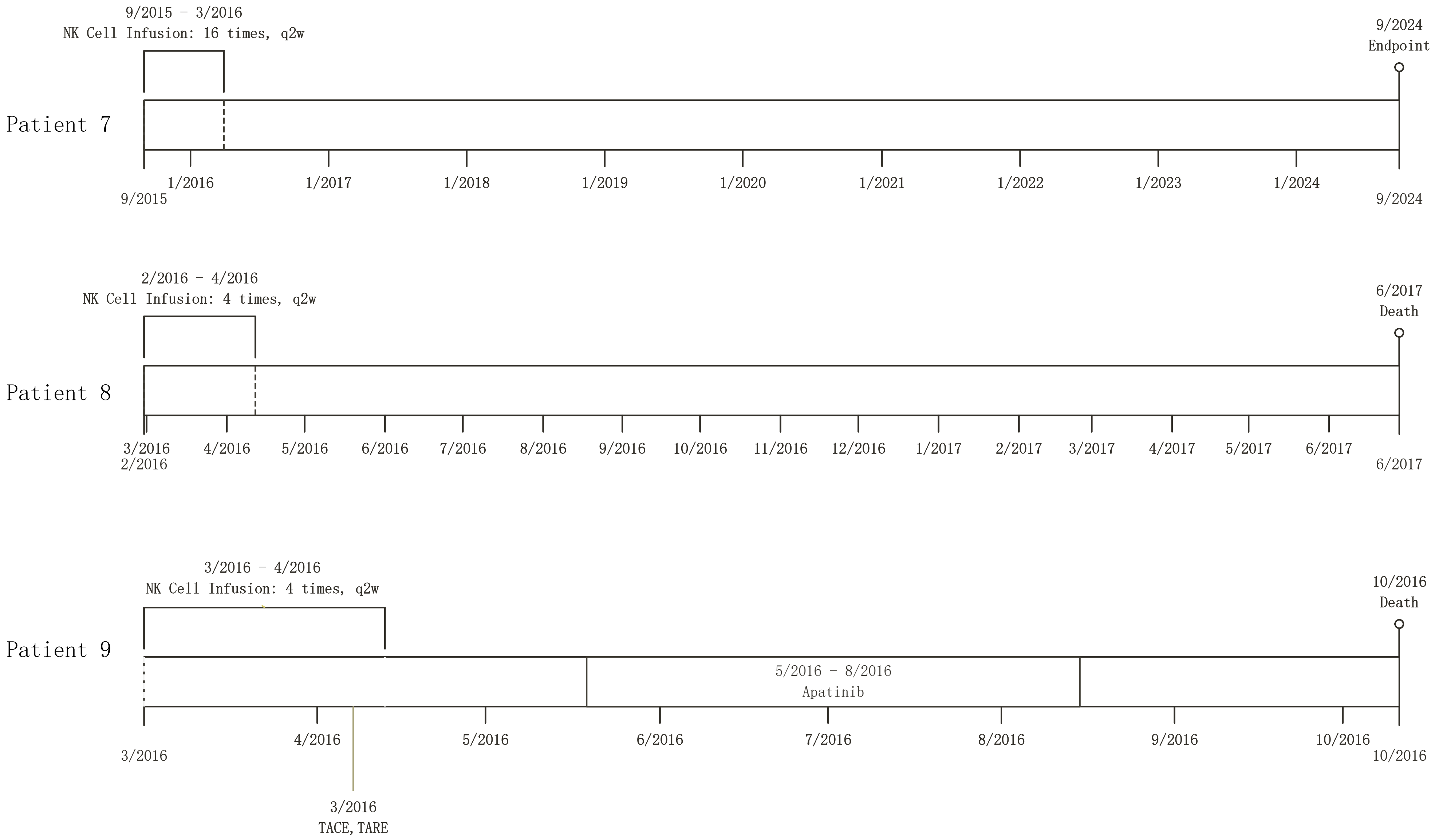
9/2015 - 3/2016
NK Cell Infusion: 16 times, q2w
9/2024
Endpoint
Patient 7
1/2016
1/2017
1/2018
1/2019
1/2020
1/2021
1/2022
1/2023
1/2024
9/2015
9/2024
2/2016 - 4/2016
NK Cell Infusion: 4 times, q2w
6/2017
Death
Patient 8
3/2016
4/2016
5/2016
6/2016
7/2016
8/2016
9/2016
10/2016
11/2016
12/2016
1/2017
2/2017
3/2017
4/2017
5/2017
6/2017
2/2016
6/2017
3/2016 - 4/2016
NK Cell Infusion: 4 times, q2w
10/2016
Death
Patient 9
5/2016 - 8/2016
Apatinib
4/2016
5/2016
6/2016
7/2016
8/2016
9/2016
10/2016
3/2016
10/2016
3/2016
TACE,TARE

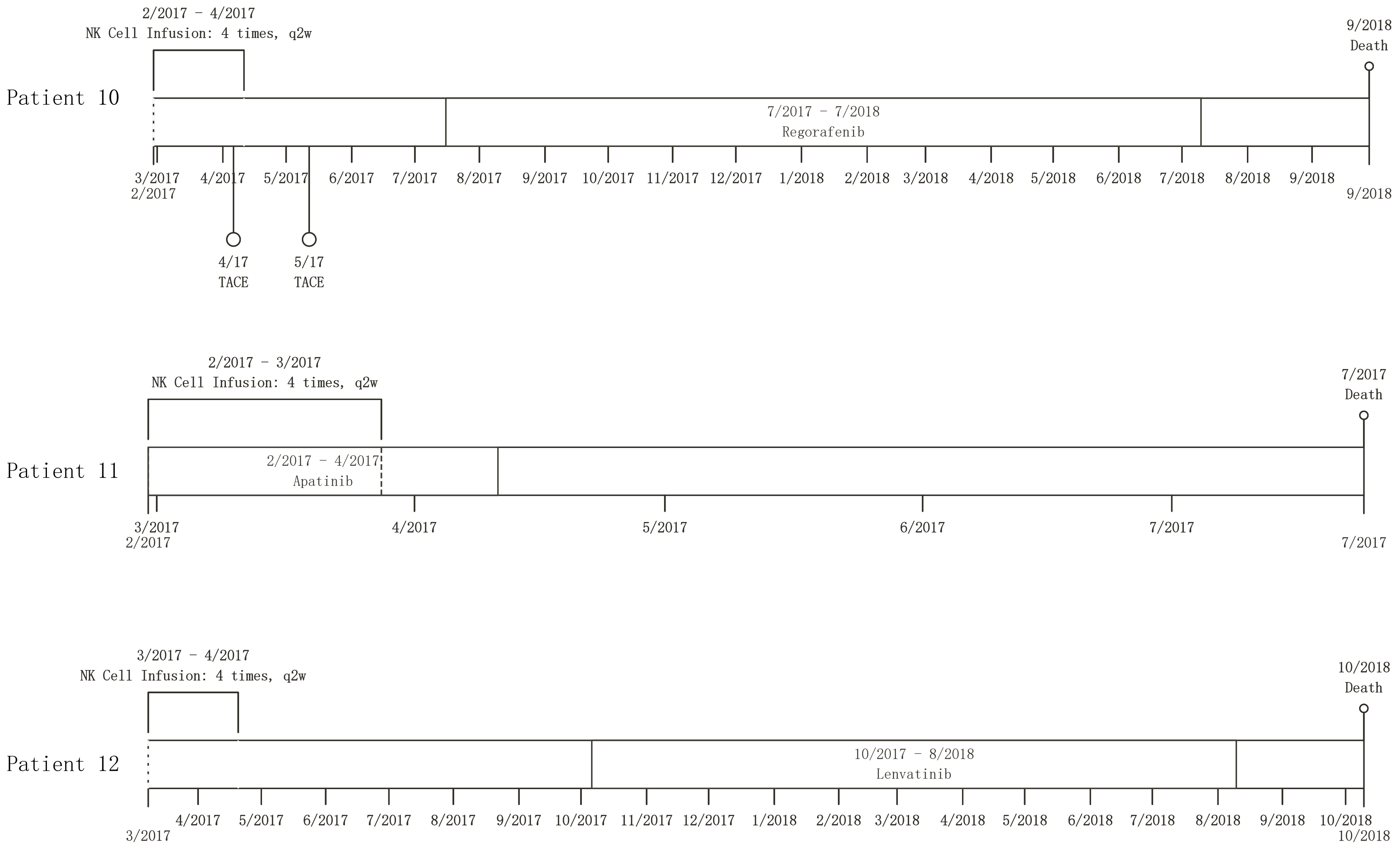
Patient 10
2/2017 - 4/2017
NK Cell Infusion: 4 times, q2w
7/2017 - 7/2018
Regorafenib
9/2018
Death
3/2017
2/2017
4/2017
5/2017
6/2017
7/2017
8/2017
9/2017
10/2017
11/2017
12/2017
1/2018
2/2018
3/2018
4/2018
5/2018
6/2018
7/2018
8/2018
9/2018
9/2018
4/17
TACE
5/17
TACE
Patient 11
2/2017 - 3/2017
NK Cell Infusion: 4 times, q2w
2/2017 - 4/2017
Apatinib
7/2017
Death
3/2017
2/2017
4/2017
5/2017
6/2017
7/2017
7/2017
Patient 12
3/2017 - 4/2017
NK Cell Infusion: 4 times, q2w
10/2017 - 8/2018
Lenvatinib
10/2018
Death
3/2017
4/2017
5/2017
6/2017
7/2017
8/2017
9/2017
10/2017
11/2017
12/2017
1/2018
2/2018
3/2018
4/2018
5/2018
6/2018
7/2018
8/2018
9/2018
10/2018
10/2018

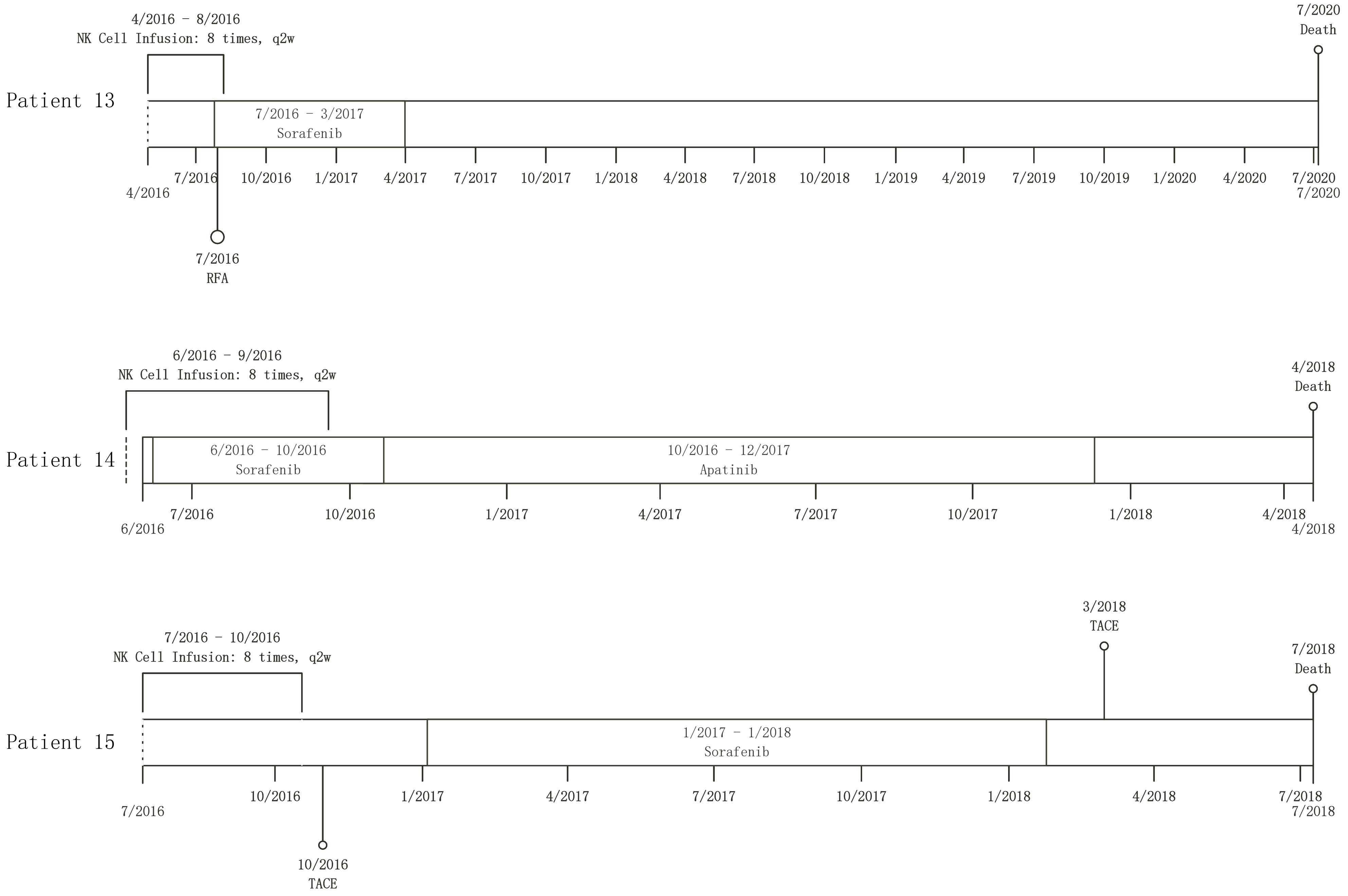
Patient 13
4/2016 - 8/2016
NK Cell Infusion: 8 times, q2w
7/2016 - 3/2017
Sorafenib
7/2016
RFA
7/2020
Death
4/2016
7/2016
10/2016
1/2017
4/2017
7/2017
10/2017
1/2018
4/2018
7/2018
10/2018
1/2019
4/2019
7/2019
10/2019
1/2020
4/2020
7/2020
7/2020
Patient 14
6/2016 - 9/2016
NK Cell Infusion: 8 times, q2w
6/2016 - 10/2016
Sorafenib
10/2016 - 12/2017
Apatinib
4/2018
Death
6/2016
7/2016
10/2016
1/2017
4/2017
7/2017
10/2017
1/2018
4/2018
4/2018
Patient 15
7/2016 - 10/2016
NK Cell Infusion: 8 times, q2w
1/2017 - 1/2018
Sorafenib
3/2018
TACE
7/2018
Death
10/2016
TACE
7/2016
10/2016
1/2017
4/2017
7/2017
10/2017
1/2018
4/2018
7/2018
7/2018

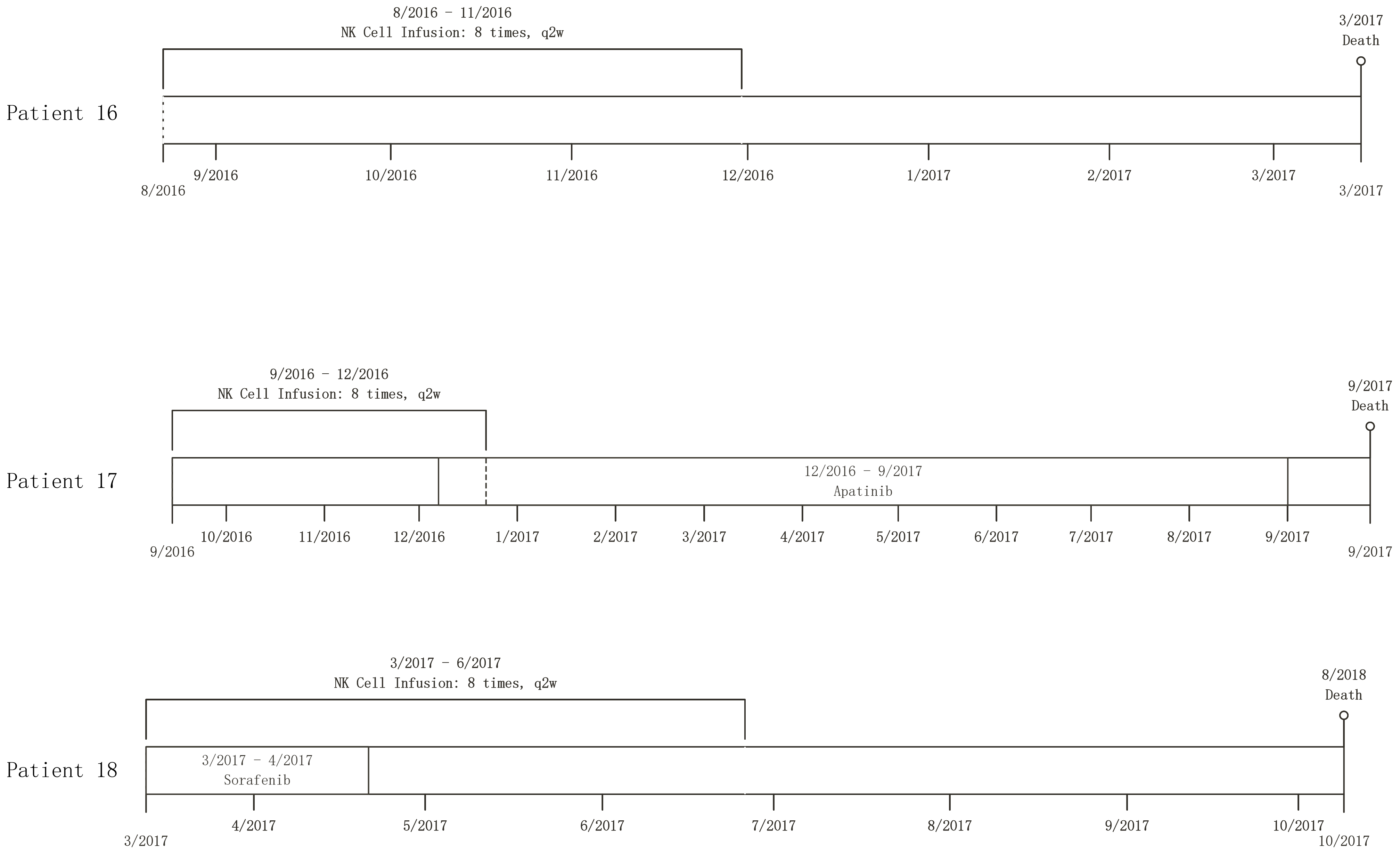

8/2016 – 11/2016
NK Cell Infusion: 8 times, q2w
3/2017
Death
Patient 16
9/2016
10/2016
11/2016
12/2016
1/2017
2/2017
3/2017
8/2016
3/2017
9/2016 – 12/2016
NK Cell Infusion: 8 times, q2w
9/2017
Death
Patient 17
12/2016 – 9/2017
Apatinib
10/2016
11/2016
12/2016
1/2017
2/2017
3/2017
4/2017
5/2017
6/2017
7/2017
8/2017
9/2017
9/2016
9/2017
3/2017 – 6/2017
NK Cell Infusion: 8 times, q2w
8/2018
Death
Patient 18
3/2017 – 4/2017
Sorafenib
4/2017
5/2017
6/2017
7/2017
8/2017
9/2017
10/2017
3/2017
10/2017

Supplementary figure 2. A schematic overview of the natural killer (NK) cell infusion schedules and key clinical events for individual patients. Horizontal bars represent the duration of NK cell therapy, with the number of infusions and interval (q2w, every two weeks) specified. Key clinical events, including surgical interventions (e.g., chest wall tumor resection and right lower lobectomy), other therapies (TACE, transarterial chemoembolization; TARE, transarterial radioembolization; RFA, radiofrequency ablation), and outcomes (mortality), are annotated along the timeline. The data illustrates the varied frequency and duration of NK cell treatment cycles administered across the patient cohort.

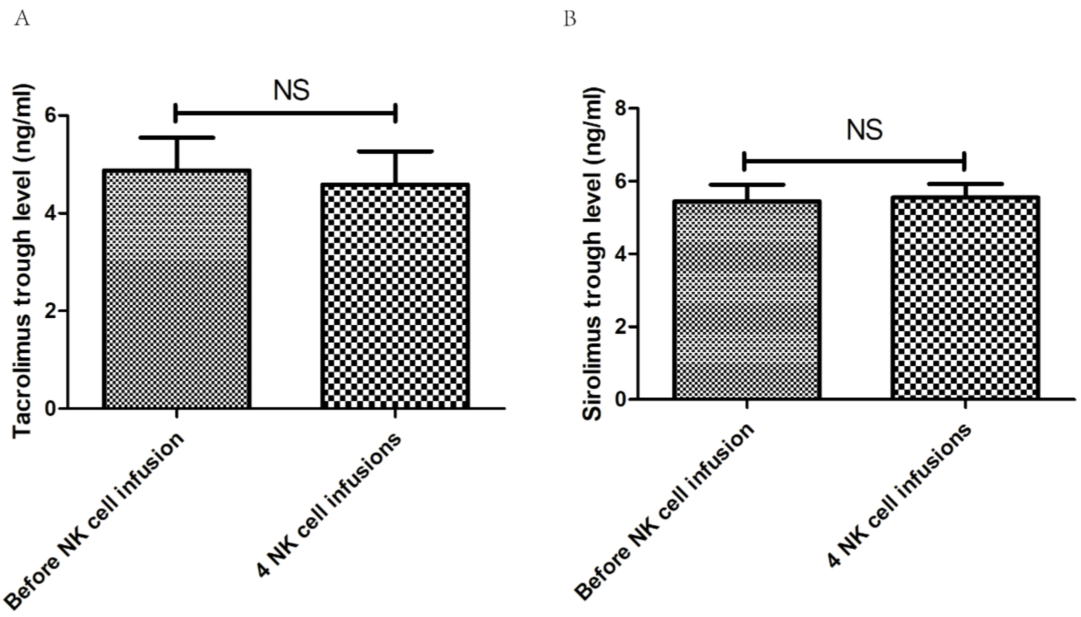

Supplementary figure 3. Trough levels of immunosuppressive drugs before and after natural killer (NK) cell infusion (n=18)..**(A)** Tacrolimus trough levels (ng/ml)before NK cell infusion and after four NK cell infusions.**(B)** Sirolimus trough levels(ng/ml) before NK cell infusion and after four NK cell infusions.Data are presented as bar graphs. Error bars represent the standard deviation or standard error. "NS" indicates that the difference between the groups is not statistically significant.
