# Supplementary material for: NK cell infusion is well-tolerated and shows preliminary efficacy in patients with recurrent hepatocellular carcinoma post-liver transplantation : a phase I trial
Source: J Transl Med. 2026 Jan 24;24:261. doi: 10.1186/s12967-026-07725-x (PMC12911381; doi:10.1186/s12967-026-07725-x)
Supplement: Supplementary file 2 — Supplementary Material 2 [file 12967_2026_7725_MOESM2_ESM.docx]

**Supplementary Table1 Demographic and Clinical Characteristics at Baseline of the Enrolled Patients**

| **Characteristic** | **A** | **B** | **C** | **D** |
| --- | --- | --- | --- | --- |
| Number of Patients | 3 | 5 | 6 | 4 |
| Age [(year), mean ± SD] Mean | 50.67±12.17 | 51.40±12.43 | 48.50±9.17 | 40.25±8.50 |
| Performance status | 1（100） | 1（100） | 1（100） | 1（100） |
| Male/Female Ratio | 3/0 | 5/0 | 6/0 | 4/0 |
| Etiology | Hepatitis B（100） | Hepatitis B（100） | Hepatitis B（100） | Hepatitis B（100） |
| Median Days of Recurrence after Liver Transplantation | 39 （27 - 147 ） | 169 （87 - 449 ） | 108 （31 - 843 ） | 147.5 （57 - 189 ） |
| BCLC stage c | 3（100） | 5（100） | 6（100） | 4（100） |
| Outside Milan criteria | 3（100） | 5（100） | 6（100） | 4（100） |
| Median Follow - up (months) | 10.8（3.8 - 12.3） | 14.3 （3.0 - 17.5） | 14.0（3.1 - 47.3） | 19.15 （14.1 - 108.1） |

• Results are reported as n (%) unless otherwise indicated.

**S****upplementary Table2. Distribution of Concomitant Anticancer Therapies Across NK Cell Dosing Groups**

| **Concomitant Therapy Modality** | **Group A**  **(n=3)** | **Group B**  **(n=5)** | **Group C**  **(n=6)** | **Group D**  **(n=4)** | **Overall**  **(n=18)** | ***P*-Value** |
| --- | --- | --- | --- | --- | --- | --- |
| **Loco-regional Therapy** |  |  |  |  |  |  |
| TACE | 0 (0.0%) | 2(40.0%) | 1( 16.7%) | 2(50%) | 5(27.8%) | 0.65 |
| RFA | 0(0%) | 0 (0%) | 1(16.7%) | 1(25%) | 2 (11.1%) | 0.50 |
| **Systemic Therapy** |  |  |  |  |  |  |
| Sorafenib | 3(100.0%) | 0(0.0%) | 2 (33.3%) | 3(75%) | 8 (44.4%) | 0.02 |
| Apatinib | 0 (0.0%) | 2(40%) | 2 (33.3%) | 1(25%) | 5 (27.8%) | 0.70 |
| **Surgical Intervention** |  |  |  |  |  |  |
| Surgical Resection | 1 (33.3%) | 0(0%) | 0 (0%) | 0 (0%) | 1 (5.6%) | 0.17 |

*Data are presented as number of patients (percentage) unless otherwise specified. P-values for categorical variables (TACE, Radiotherapy, Sorafenib, Apatinib, Surgical Resection) were calculated by Fisher’s exact test. TACE: transcatheter arterial chemoembolization, RFA: radiofrequency ablation,

**Supplementary Table 3. Exploratory correlation analysis between baseline characteristics and survival outcomes**

| **Analysis** | **n** | **Spearman‘s ρ** | **P value** |
| --- | --- | --- | --- |
| Age vs. PFS | 18 | -0.414 | 0.088 |
| Age vs. OS | 18 | -0.434 | 0.072 |
| Days to Recurrence (post-LTx) vs. PFS | 18 | 0.148 | 0.559 |
| Days to Recurrence (post-LTx) vs. OS | 18 | 0.416 | 0.086 |

* Days to Recurrence is measured from the date of liver transplantation (LTx) to the date of radiological recurrence, while PFS and OS in this study are measured from the start date of NK cell therapy. A significant correlation, if observed, may reflect the underlying aggressiveness of the disease rather than a direct relationship with the efficacy of NK cell therapy.Spearman's rank correlation coefficient (ρ) was used to assess monotonic relationships. All analyses are exploratory due to small sample size.

## **Supplementary Table 4. Exploratory correlation analysis between NK cell product purity and survival outcomes**

| **Analysis** | **n** | **Spearman‘s ρ** | **P value** |
| --- | --- | --- | --- |
| **NK cell** Purity vs. PFS | 18 | -0.131 | 0.604 |
| **NK cell** Purity vs. OS | 18 | -0.069 | 0.785 |

* Purity refers to the percentage of CD56⁺CD3 cells in the final infusion product. Analysis includes data from all infusion batches. Spearman's rank correlation coefficient (ρ) was used to assess monotonic relationships. All analyses are exploratory due to small sample size.

**Supplementary Table 5. Exploratory multivariable Cox regression analysis for OS**

| **Variable** | **Category** | **Hazard Ratio**  **(HR)** | **95% Confidence Interval**  **(CI)** | **P value** |
| --- | --- | --- | --- | --- |
| Treatment Group |  |  |  | 0.044 |
|  | Group A(Reference) |  |  |  |
|  | Group B | 1.102 | 0.212 – 5.724 | 0.908 |
|  | Group C | 0.401 | 0.066 – 2.418 | 0.319 |
|  | Group D | 0.027 | 0.002 – 0.382 | 0.008 |
| **Age** | Per year increase | 1.024 | 0.967 – 1.083 | 0.419 |
| Time to Recurrence | Per day increase | 0.997 | 0.993 – 1.000 | 0.056 |

* CI： confidence interval; OS： overall survival. This exploratory analysis is based on a small sample (n=18), resulting in wide confidence intervals.
