# Supplementary material for: NK cell infusion is well-tolerated and shows preliminary efficacy in patients with recurrent hepatocellular carcinoma post-liver transplantation : a phase I trial
Source: J Transl Med. 2026 Jan 24;24:261. doi: 10.1186/s12967-026-07725-x (PMC12911381; doi:10.1186/s12967-026-07725-x)
Supplement: Supplementary file 3 — Supplementary Material 3 [file 12967_2026_7725_MOESM3_ESM.docx]

Dear Editors of Journal of Translational Medicine,

I am pleased to submit our manuscript entitled “NK Cell Infusion is Well-Tolerated and Effective in Patients with Recurrent Hepatocellular Carcinoma Post-Liver Transplantation” for consideration for publication in Journal of Translational Medicine.

This study builds upon our previous work published in Hepatology International (Chen et al., 2023, DOI: https://doi.org/10.1007/s12072-023-10524-x), which established the safety of TCR-redirected T-cell therapy for HBV-related hepatocellular carcinoma (HCC) recurrence after liver transplantation. In the present research, we address a critical unmet need in this high-risk patient population by evaluating natural killer (NK) cell-based immunotherapy as a novel therapeutic strategy.

Our findings demonstrate that adjunctive NK cell infusion is well-tolerated, with minimal adverse events, and significantly improves both progression-free survival (PFS) and overall survival (OS) in patients with recurrent HCC following liver transplantation. Importantly, we identify an incremental dosing schedule associated with optimal clinical outcomes, suggesting a meaningful dose-frequency relationship that may inform future therapeutic protocols. These results position NK cell therapy as a promising intervention for this challenging clinical scenario.

Given Journal of Translational Medicine’s dedication to bridging basic research and clinical application, we believe our study aligns closely with the journal’s mission by providing:

1）The first clinical evidence supporting the efficacy of NK cell therapy in preventing HCC recurrence post-transplantation.

2）Long-term safety and survival validation through a 9-year follow-up, offering a valuable benchmark for future translational studies in oncology.

3）An optimized dosing paradigm based on clinical outcomes, tailored for high-risk immunosuppressed patients.

4）Novel mechanistic insights into immunotherapy approaches in transplant recipients, addressing a key gap in translational immuno-oncology.

We confirm that this manuscript represents original work, has not been published previously, and is not under consideration elsewhere. All authors have approved the submission and declare no conflicts of interest.

We are confident that the translational relevance of this research could influence therapeutic strategies in transplant oncology and would be happy to provide any additional data or revisions as required.

Thank you for your time and consideration.

Sincerely,

Corresponding author:

Wenjie Chen

Email: [chenwj5@mail.sysu.edu.cn](mailto:chenwj5@mail.sysu.edu.cn)
