# Supplementary material for: NK cell infusion is well-tolerated and shows preliminary efficacy in patients with recurrent hepatocellular carcinoma post-liver transplantation : a phase I trial
Source: J Transl Med. 2026 Jan 24;24:261. doi: 10.1186/s12967-026-07725-x (PMC12911381; doi:10.1186/s12967-026-07725-x)
Supplement: Supplementary file 4 — Supplementary Material 4 [file 12967_2026_7725_MOESM4_ESM.docx]

Dear Editors,

Thank you for granting us the opportunity to submit the revised draft of our manuscript entitled " NK Cell Infusion is Well-Tolerated and Shows Preliminary Efficacy in Patients with Recurrent Hepatocellular Carcinoma Post-Liver Transplantation : A Phase I Trial" (JTRM-D-25-21570) for publication in the Journal of Translational Medicine. We greatly appreciate the time and effort you and the reviewers have dedicated to reviewing our work and providing constructive feedback—your insightful comments have been invaluable for refining our manuscript.

We have addressed and incorporated all actionable suggestions from the reviewers, and these revisions are highlighted in red within the manuscript. Below is our point-by-point response to all reviewers’ comments and concerns, with our responses highlighted in red. All page numbers cited here in correspond to the revised manuscript file (marked with track changes).

Please don't hesitate to contact me if you have any further questions.

 Thank you and best regards.

Yours sincerely,

Corresponding author: Wenjie Chen

E-mail: [chenwj5@mail.sysu.edu.cn](mailto:chenwj5@mail.sysu.edu.cn)

Replies to the reviewers' comments:

Reviewer #1:

- Title: I prefer to re-write the title of this article as follows: "Perspectives on NK cell therapy for the future of HCC treatment".

**Response:** Thank you for your suggestion regarding the title. We appreciate the reviewer’s perspective and the opportunity to refine our title to better reflect the nature and scope of this work.

We have carefully considered the proposed title, “Perspectives on NK cell therapy for the future of HCC treatment.” While we agree that discussing the future implications of NK cell therapy is valuable, this phrasing is typically used for review or opinion articles that synthesize existing literature and outline future directions. Our manuscript, in contrast, reports original findings from a Phase I clinical trial. Therefore, a descriptive title that directly conveys the study’s design, population, and key outcomes is more appropriate for a research article.

To address the reviewer’s underlying concern—that the title should accurately communicate the preliminary nature of our findings—we have revised the title to:

“NK Cell Infusion is Well-Tolerated and Shows Preliminary Efficacy in Patients with Recurrent Hepatocellular Carcinoma Post-Liver Transplantation : A Phase I Trial”

This revised title:

1）Clearly indicates the study is a Phase I trial, setting appropriate expectations for its exploratory nature.

2）Uses cautious language (“Shows Preliminary Efficacy”) to reflect the preliminary evidence generated.

3）Retains the essential descriptive elements (intervention, patient population, and context) necessary for readers to immediately understand the article’s focus.

- Just a simple inquiry

- What side effects do NK cell infusions frequently cause?

- How does NK cell therapy impact survival in HCC patients?

**Response:** Thank you for these excellent and direct questions regarding the core findings of our study. We are pleased to provide the following clarifications based on our trial data:

1. What side effects do NK cell infusions frequently cause?
The most frequently observed treatment-related side effect in this study was low-grade fever (Grade 1 pyrexia), which occurred in 7 out of 18 patients (38.9%). This fever typically developed shortly after infusion and resolved spontaneously within one day. No severe or dose-limiting toxicities (such as ≥ Grade 3 non-hematological or Grade 4 hematological toxicities) were attributed to the NK cell infusions. Critically, we observed no cases of graft rejection, significant liver dysfunction, or viral reactivation related to the treatment, supporting the favorable safety and tolerability profile of this therapy in post-transplant patients.

2. How does NK cell therapy impact survival in HCC patients?
In this exploratory Phase I trial, we observed a potential dose-response trend related to survival outcomes. Patients receiving higher cumulative doses and more frequent infusions (particularly Group D on an incremental schedule) demonstrated numerically longer median survival times:

1）Progression-Free Survival (PFS): 7.5 months for Group D vs. 1.6–5.5 months for other groups.

2）Overall Survival (OS): 31.3 months for Group D vs. 12.6–18.4 months for other groups.

This suggests that the intensity and schedule of NK cell therapy may be associated with improved survival in this challenging patient population with recurrent HCC after liver transplantation. We wish to emphasize that these findings are preliminary and hypothesis-generating, given the study's small sample size and non-randomized design. The results strongly support the need for further investigation in larger, controlled trials to confirm these initial observations and define the optimal therapeutic regimen.

Reviewer #2:

1. Abstract:

The language in the Abstract should be tempered to accurately reflect the exploratory, hypothesis-generating nature of a Phase I study. Phrases like "significantly influenced" imply a definitive causal effect not established by this small, uncontrolled trial. Suggest rephrasing to more cautious language such as "was associated with improved," "suggests a positive impact on," or "indicates a potential dose-response relationship."

Response: Thank you for this valuable comment. We agree entirely that the language in the Abstract should precisely reflect the exploratory nature of our Phase I trial. As suggested, we have carefully revised the manuscript to replace definitive terms like "significantly influenced" with " associated with differences in" to accurately frame our findings(Page 2, Lines 46).

Furthermore, to explicitly state the purpose of these analyses, we have clarified in the Methods and Results sections of the Abstract that PFS and OS were assessed as exploratory endpoints to evaluate preliminary antitumor activity.This wording underscores that the efficacy analyses were hypothesis-generating, intended to inform the design of future studies rather than to establish definitive conclusions. (Page 2, Lines 25–31).

2. Introduction:

1）The introduction is currently lengthy and contains background details that, while relevant, could be streamlined. It is recommended to condense the epidemiological and basic biology sections to provide a more focused rationale.

2）The introduction should efficiently establish the unmet need in managing post-transplant HCC recurrence, briefly summarize the therapeutic rationale for NK cells in this setting, and clearly state the specific research gap this Phase I trial aims to fill.

**Response:** Thank you for the suggestion. We have condensed the Introduction to improve focus and flow, reducing excessive epidemiological and basic biology details

To make the introduction more concise, we have removed detailed descriptions of the molecular mechanisms underlying NK cell-mediated cytotoxicity, including:The specific process of pore formation by perforin (~16 nm pores).The role of granzymes in activating caspase-3 and caspase-10 to induce apoptosis.The involvement of granulysin (GNLY) and TNF-related ligands (TRAIL and FasL) in apoptotic pathways.Cytokine production (IFN-γ and TNF-α) and antibody-dependent cellular cytotoxicity (ADCC).These details, while informative, are more suited to a specialized immunology context. Their removal helps maintain focus on the clinical rationale and study objectives of this Phase I trial. (Page5, Lines 86–95).

3. Methods:

1） Clarity on "Conventional Treatment": The term "conventional treatment" is used throughout but remains vague. Please provide a clear, concise definition early in the Methods section (e.g., in a dedicated subsection) detailing the standard-of-care therapies patients received concurrently with NK cell infusions.

2）The description of the NK cell culture process would benefit from greater clarity regarding the timeline. Please specify key timepoints and the duration of each major culture phase.

3）Briefly reiterate the donor eligibility criteria (healthy relatives, ABO-matched) in the manufacturing subsection for completeness.

**Response:** Thank you for your comment. 1）We have added a dedicated subsection to define “conventional treatment,” clarified the NK cell culture timeline, and reiterated donor eligibility in the manufacturing section. (Page 7, Lines 147–150).

2）The isolated PBMCs were subsequently cultured in vitro for 14 days.(Page 8, Lines 153–154).

3) Peripheral blood (30–60 ml) was collected from patients' healthy relatives by matching ABO blood types to isolate peripheral blood mononuclear cells (PBMCs).(Page 8, Lines 152–153).

4. Results:

Ensure that Table 2 and the results text explicitly state that the listed concomitant therapies were administered during the NK cell treatment period.

**Response:** We thank you for this constructive suggestion. We have revised Table 2 and its corresponding textual references to explicitly clarify that concomitant therapies were administered during the NK cell infusion period.Key modifications are summarized below:

1）Table 2 title revised from "Baseline characteristics of patients receiving NK-cell treatment" to "Baseline characteristics and concomitant therapies during NK cell treatment period".

2）A supplementary footnote added to Table 2: All concomitant anti-carcinoma and immunosuppressive therapies listed were administered during the NK cell infusion period.

5. Discussion:

1） Sharpened Novelty Statement: Begin the discussion by more explicitly stating the core innovation of this work (e.g., the first trial using relative-derived, ex vivo expanded allogeneic NK cells for this specific indication).

2）The point that NK cells are less likely to cause severe GVHD or rejection is a key strength. To substantiate this, please cite 1-2 key references discussing the relatively favorable safety profile of allogeneic NK cell infusions in relevant settings.

3）The discussion on why Group D showed the best outcomes is brief. Please expand with plausible hypotheses

4）More deeply address the confounding factor of heterogeneous concomitant therapies. Elaborate on how this affects the interpretation of the survival data, explicitly acknowledging that efficacy signals may stem from the combined therapeutic approach.

**Response:** Thank you for the suggestion.We have revised the Discussion to address each of these points thoughtfully.

1）The adoptive transfer of ex vivo-expanded, relative-derived allogeneic NK cells was well-tolerated in this Phase I dose-escalation study, which is the first to evaluate this therapeutic approach for recurrent HCC following liver transplantation.(Page 17, Lines 349–351).
2）Moremover，NK cells are less likely to cause severe GVHD or rejection is a key strength^【42】^ (Page 20, Lines431–432).To substantiate this key advantage, we have cited a pivotal reference demonstrating the favorable safety profile of allogeneic NK cell infusions:

【42】Ruggeri L, Capanni M, Urbani E, Perruccio K, Shlomchik WD, Tosti A, et al. Effectiveness of donor natural killer cell alloreactivity in mismatched hematopoietic transplants. Science 2002, 295(5562): 2097-2100.

3）Expanded on Group D outcomes: Added mechanistic hypothesis: The incremental dosing schedule in Group D may have allowed for gradual immune engagement, reducing early cytokine-related toxicity while promoting sustained NK cell activity and adaptive immune priming.”(Page 19, Lines 412–414).

4)The small, non-randomized sample size and the heterogeneity of concomitant anticancer treatments received by patients are considerable limitations that preclude definitive conclusions on clinical efficacy. Although our post hoc analysis indicated no significant differences in the distribution of major concomitant therapies across groups, we cannot entirely rule out their potential influence as confounding factors.

6. Conclusions:

The conclusion must be carefully framed to align with the Phase I data. It should reiterate the promising safety and preliminary efficacy signals, emphasize the observed dose-response trend, and strongly underscore the necessity for future larger-scale, randomized controlled trials for validation.

**Response:** To temper the conclusions in line with the exploratory nature of our study, we have revised the final paragraph. The definitive claim that strategies “significantly impact survival” has been moderated to state they “influence survival outcomes”. Most importantly, we have inserted the key sentence, “Therefore, they should be considered hypothesis-generating,” to clearly frame the findings as preliminary(Page20, Lines 426–432).Future multicenter randomized controlled trials should be designed to further investigate the effectiveness of NK cell therapy in patients with recurrent HCC after liver transplantation.(Page 22, Lines 463–470).

Reviewer #3:

1. The Methods section states that "Utilizing the random number table method, all eligible patients were randomly stratified into four distinct groups," yet the Strengths and Limitations section acknowledges "the small, non-randomized sample size." This fundamental contradiction requires clarification. Furthermore, Supplementary Figure 2 shows a sequential enrollment pattern (DABC order) that is inconsistent with proper randomization procedures. The authors must either provide documentation of the randomization process or revise all relevant text to accurately reflect the actual study design.

**Response:** Thank you for identifying this critical inconsistency, which was a result of an error in our original text. We have corrected this throughout the manuscript. The study was a non-randomized, multi-arm dose/schedule-finding Phase I trial. Patient allocation to the four treatment arms was sequential and guided by a predefined protocol to explore different dose-frequency combinations, not by randomization.The sequential pattern (D-A-B-C) reflected the order in which the protocol arms were opened for enrollment to concurrently explore these distinct regimens, not a randomization sequence.

In the Methods section, we have revised the relevant text as follows: Eligible patients were allocated into four treatment groups based on a predefined dose-escalation and schedule-expansion scheme, rather than through randomization. The assignment aimed to explore different dose-frequency combinations and was conducted sequentially as patients were enrolled (Page 6, Lines 122–126).

2. The study enrolled patients in D-A-D-B-C order (starting with the highest dose and longest duration group D, followed by progressively lower dose/shorter duration groups). This approach contradicts standard dose-escalation trial principles where safety should first be established with lower doses before proceeding to higher doses. The authors must justify this unusual enrollment strategy and address how potential temporal biases were controlled for.

Response：Thank you for correctly identifying this deviation from the conventional "3+3" dose-escalation design. Our trial was designed as a parallel dose/schedule exploration study rather than a single-agent dose-escalation study. The starting dose (5×10⁹ cells) for Groups A and the initial phase of Group D was selected based on prior safety data of allogeneic NK cells in other settings and was considered a safe starting point. The design aimed to concurrently evaluate the feasibility, safety, and preliminary activity of several distinct therapeutic strategies (varying both total dose and frequency), with Group D representing a more complex "induction-maintenance" paradigm.

We have explicitly acknowledged this design choice and its corresponding implications in the Discussion section, where we have added the relevant text as follows: Second, the study employed a non-randomized, multi-arm design with sequential enrollment into different dose-frequency regimens, rather than a traditional dose-escalation schema. This approach, while enabling the exploration of multiple strategies, may introduce temporal or selection biases that cannot be fully adjusted for in the analysis."(Page 20, Lines 433–436).

To controlling for temporal bias，in our analysis, all survival outcomes (PFS and OS) were calculated from the start date of NK cell therapy, not the date of enrollment or transplantation, to minimize bias from differences in enrollment time. However, we acknowledge this cannot fully eliminate all temporal biases.

3. Table 1 reveals significant baseline imbalances between groups, particularly the younger age in group D and earlier recurrence after transplantation in group A compared to other groups. These differences could substantially impact survival outcomes independent of the NK cell intervention. The authors should explain the criteria used for patient allocation to different groups and perform analyses to account for these baseline differences.

Response：Thank you for your suggestion.As clarified in response to Comment 1, allocation was non-random and sequential. The criteria were primarily the availability of a slot in the predefined treatment arm at the time of patient enrollment and the patient's willingness/ability to adhere to the specific regimen (e.g., the longer commitment required for Group D).

We have added the relevant text to the Results sections as follows:We performed Spearman correlation tests across the entire cohort (n=18) between the continuous baseline variables "Age" and "Days to Recurrence" and the survival endpoints PFS and OS. The results showed no statistically significant correlations (all p > 0.05) in this small data set. These results are presented in the new Supplementary Table 3 and mentioned in the revised Results section(Page 15-16, Lines 327–330)..

Supplementary Table 3. Exploratory analysis of potential confounding variables

| **Analysis** | **n** | **Spearman‘s ρ** | **P value** |
| --- | --- | --- | --- |
| Age vs. PFS | 18 | -0.414 | 0.088 |
| Age vs. OS | 18 | -0.434 | 0.072 |
| Days to Recurrence (post-LTx) vs. PFS | 18 | 0.148 | 0.559 |
| Days to Recurrence (post-LTx) vs. OS | 18 | 0.416 | 0.086 |

* Days to Recurrence is measured from the date of liver transplantation (LTx) to the date of radiological recurrence, while PFS and OS in this study are measured from the start date of NK cell therapy. A significant correlation, if observed, may reflect the underlying aggressiveness of the disease rather than a direct relationship with the efficacy of NK cell therapy.

In addition,we have strengthened the Limitations section: "Third, the non-randomized allocation resulted in baseline imbalances between groups. Although post-hoc correlation analyses in our small cohort did not show significant associations between these specific variables (age, time to recurrence) and survival outcomes, their potential confounding influence cannot be entirely excluded."(Page 20, Lines 436–439).

4.Different NK cell donors (NK cells derived from the peripheral blood of relatives) were used for different patient groups, introducing a significant confounding variable. Without evidence demonstrating functional equivalence between NK cell products from different donors, it is impossible to determine whether observed outcome differences relate to the treatment regimen or donor-specific product characteristics. The authors should provide functional potency data (e.g., cytotoxicity assays) for all administered products to establish comparability.

Response：Thank you for your comment. This is indeed a very valid point. We acknowledge that donor-to-donor variability is a known and important factor in allogeneic cell therapy.We must clarify that standardized, batch-wise functional potency assays (e.g., cytotoxicity against tumor cell lines) were not performed on the final infusion products as part of the release criteria for this early-phase trial. The primary focus was on establishing safety and feasibility using products meeting defined quality control (QC) release criteria (viability >90%, CD56^+^CD3^-^ purity >80%, sterility, endotoxin levels).We have added a clear statement to the Discussion (Limitations): "Fourth, NK cells were derived from different related donors without HLA/KIR matching. While all products met predefined release criteria, we did not perform standardized functional potency assays across all batches. Therefore, potential donor-to-donor variability in NK cell functional activity remains an unmeasured confounder that could have influenced outcomes."(Page 21, Lines 446–4509).

5.While the authors cite "CD56⁺CD3⁻ cells > 80%" as a quality parameter, Supplementary Figure 1 shows substantial variation in this metric across batches (ranging from 80% to nearly 100%). This variability could significantly impact therapeutic efficacy. The authors should analyze whether NK cell purity correlates with clinical outcomes or provide additional evidence that product variability did not influence results. In addition, the T cell contamination is critic for the safety of allogeneic NK cell infusion, therefore, the percentage of T cells must be provided.

**Response:**Thank you for raising these critical points regarding product characterization and its potential impact on outcomes. We have performed the suggested analyses and provided the requested data, as detailed below.As suggested, we performed an exploratory Spearman correlation analysis to assess whether the purity of the infused NK cell products (percentage of CD56^+^CD3^-^cells) was associated with patient survival. The analysis, which included data from all infusion batches, found no statistically significant correlation between product purity and either Progression-Free Survival (PFS) or Overall Survival (OS) (Spearman's ρ for PFS: [ρ value= -0.131, P= 0.604]; for OS: [ρ value= -0.069, P= 0.785]).

**Supplementary Table 4.** Exploratory correlation analysis between NK cell product purity and survival outcomes

| **Analysis** | **n** | **Spearman‘s ρ** | **P value** |
| --- | --- | --- | --- |
| NK cell Purity vs. PFS | 18 | -0.131 | 0.604 |
| NK cell Purity vs. OS | 18 | -0.069 | 0.785 |

* Purity refers to the percentage of CD56^+^CD3^-^cells in the final infusion product. Analysis includes data from all infusion batches. Spearman's rank correlation coefficient (ρ) was used to assess monotonic relationships. All analyses are exploratory due to small sample size.

We fully agree on the importance of reporting T-cell contamination for safety assessment. As requested, we have analyzed and now report the level of CD3⁺ T-cell contamination in all final infusion products. The data are summarized in the new Supplementary Figure 1B，As shown, the level of T-cell contamination was consistently low in all groups and for all patients.This low and consistent T-cell level aligns with the absence of any GVHD or graft rejection events observed in our study, supporting the safety profile of the manufacturing process.

The corresponding content has been added to the Results section (Page 10, Lines 212–213) and the Discussion section (Page 21, Lines 450–458).

**Supplementary figure 1. Phenotypic characterization of expanded cells from different groups.**(A)Proportion of CD56⁺CD3⁻ (NK) cells. (B)Proportion of CD3⁺(T) cells. Data are presented as scatter plots showing the percentage for each donor, with the horizontal line representing the median. Group A: n = 12, Group B: n = 20, Group C: n = 48, Group D: n = 64.

6.The survival analyses do not account for significant baseline differences between groups (age, time to recurrence, concomitant therapies). Without multivariate analyses controlling for these confounders, the claim that NK cell infusion strategies significantly impacted survival outcomes is unsupported. The authors should perform Cox regression analyses incorporating relevant covariates to isolate the effect of the NK cell intervention.

**Response：**Thank you for the essential suggestion to perform a multivariable analysis to control for potential confounders. In direct response, we conducted an exploratory multivariable Cox regression for Overall Survival, adjusting for age and time to recurrence, with Group A as the reference. The results, detailed in the new Supplementary Table 5, indicate that after adjustment, treatment group remained a significant predictor of survival (omnibus p=0.044). Specifically, Group D showed a significantly lower hazard of death compared to Group A (HR=0.027, 95% CI: 0.002–0.382, p=0.008), while the effects of age (p=0.419) and time to recurrence (p=0.056) were not significant. We have revised the Methods and Results sections to describe this analysis and have added a cautionary note in the Limitations regarding the instability of estimates due to the small sample size (n=18) and the remaining confounding from heterogeneous concomitant therapies. This analysis provides adjusted, hypothesis-generating support for a dose-response relationship, underscoring the need for future randomized trials.

**Supplementary Table 5. Exploratory multivariable Cox regression analysis for OS**

| **Variable** | **Category** | **Hazard Ratio**  **(HR)** | **95% Confidence Interval**  **(CI)** | **P value** |
| --- | --- | --- | --- | --- |
| Treatment Group |  |  |  | 0.044 |
|  | Group A(Reference) |  |  |  |
|  | Group B | 1.102 | 0.212 – 5.724 | 0.908 |
|  | Group C | 0.401 | 0.066 – 2.418 | 0.319 |
|  | Group D | 0.027 | 0.002 – 0.382 | 0.008 |
| **Age** | Per year increase | 1.024 | 0.967 – 1.083 | 0.419 |
| Time to Recurrence | Per day increase | 0.997 | 0.993 – 1.000 | 0.056 |

* CI： confidence interval; OS： overall survival. This exploratory analysis is based on a small sample (n=18), resulting in wide confidence intervals.

In response to your comment, we have revised the manuscript to incorporate the multivariable Cox regression analysis. Specifically, (1) a detailed description of the model has been added to the Methods section (Page 10, Lines 201–203).

, and (2) a new paragraph summarizing the results, with reference to Supplementary Table 5, has been included in the Results section (Page 16, Lines 339–347).

.

7.The conclusion that NK cell infusion strategies "significantly impact survival outcomes" is not justified by the presented data. Given the small sample size, baseline imbalances, heterogeneous concomitant treatments, and lack of multivariate analysis, this definitive language overinterprets exploratory findings. The conclusions should be tempered to reflect the preliminary, hypothesis-generating nature of this study.

**Response:** Thank you for your comment. We fully agree and have revised the language throughout the manuscript to reflect the preliminary nature of the findings.

**Title:** Changed to” NK Cell Infusion is Well-Tolerated and Shows Preliminary Efficacy in Patients with Recurrent Hepatocellular Carcinoma Post-Liver Transplantation : A Phase I Trial”.

**Abstract:** Replaced "significantly influenced" with " associated with differences in".could be key repalce to merits further investigation.

Furthermore, to explicitly state the purpose of these analyses, we have clarified in the Methods and Results sections of the Abstract that PFS and OS were assessed as exploratory endpoints to evaluate preliminary antitumor activity.This wording underscores that the efficacy analyses were hypothesis-generating, intended to inform the design of future studies rather than to establish definitive conclusions. (Page 2, Lines 25–31).

**Conclusions：**To temper the conclusions in line with the exploratory nature of our study, we have revised the final paragraph. The definitive claim that strategies “significantly impact survival” has been moderated to state they “influence survival outcomes”. Most importantly, we have inserted the key sentence, “Therefore, they should be considered hypothesis-generating,” to clearly frame the findings as preliminary(Page22, Lines 464–470).

.

8.Table 4 reports only one treatment-related adverse event (TRAE), which is inconsistent with expectations for cellular therapy. A comprehensive table of all treatment-emergent adverse events (TEAEs) across all groups should be provided, including severity grading and relationship to study treatment.

**Response:** Thank you for raising this important point. We would like to clarify that Table 3 already provides a detailed, patient-level account of all pyrexia events considered related to NK cell infusion, including their severity (Grade 1 or 2), duration, timing of onset, and association with specific infusion numbers and dose levels. This demonstrates that multiple such events were documented and appropriately graded.

We agree, however, that a consolidated summary of all treatment-emergent adverse events (TEAEs) across the study groups—irrespective of assessed causality—would provide a clearer overall safety profile. As outlined in the Methods and Results, we prospectively monitored for a predefined set of events of special interest in this liver transplant population receiving immunotherapy, including liver function abnormalities, viral reactivation, graft rejection, and sepsis. The consistent absence of these monitored events across all patients is itself a notable safety observation.

In summary, the relatively narrow spectrum of treatment-related adverse events, dominated by low-grade pyrexia, together with the absence of expected transplant- or immunotherapy-related complications such as rejection or significant hepatotoxicity, represents a key safety finding of our study. This is discussed in the context of the favorable safety profile of allogeneic NK cell therapy in immunosuppressed recipients.

To provide a clearer and more comprehensive summary of treatment-emergent safety data, we have added clarifying text in both the Methods and Results sections as follows:

Methods：All such events, irrespective of their assessed causality to the NK cell infusion, were documented as treatment-emergent adverse events (TEAEs). We prospectively monitored for this predefined set of events of special interest (including liver function abnormalities, HBV reactivation, allograft rejection, and sepsis) to comprehensively capture the safety profile in this unique patient population.(Page9, Lines 187–191).

Results：Collectively, the data presented in Tables 3 and 4 provide a comprehensive overview of the treatment-emergent safety profile. The spectrum of adverse events directly associated with NK cell infusion was narrow, primarily consisting of transient, low-grade pyrexia. More importantly, prospective monitoring for pre-specified events of special interest in this transplant population—including liver injury, viral reactivation, allograft rejection, and severe infection—confirmed the absence of such complications. This favorable safety observation, occurring alongside stable immunosuppressive drug levels (Supplementary Figure 3), strongly supports the tolerability of allogeneic NK cell therapy in liver transplant recipients.(Page13, Lines 277–283).

9. As a translational clinical study, the lack of NK cell persistence data, expansion kinetics, or functional biomarkers significantly limits mechanistic interpretation of the clinical outcomes. The addition of these data would substantially strengthen the manuscript's translational impact.

**Response:** Thank you for your comment. We acknowledge this as a major limitation of our current study. The primary endpoints were clinical safety and efficacy. We have added a dedicated paragraph in the Discussion (Limitations):"Sixth, this study lacks pharmacokinetic and pharmacodynamic data on the infused NK cells, such as their in vivo persistence, expansion kinetics, or functional biomarkers. Consequently, we cannot elucidate the mechanistic basis for the observed clinical outcomes. Incorporating such translational endpoints in future studies is crucial to understanding the biology of NK cell therapy in this setting."(Page21, Lines 458–462).

10.Patients received highly variable anticancer treatments alongside NK cell infusions (Table 1), which likely had substantial impact on outcomes. The authors should analyze the potential interactions between standard therapies and NK cell treatment or more thoroughly acknowledge this limitation.

**Response:** We sincerely thank the reviewer for highlighting this important limitation. We fully acknowledge that the heterogeneous concomitant anticancer therapies could confound the survival outcomes. To address this concern, we have performed additional quantitative analyses, summarized in the new Supplementary Table 5, and have revised the manuscript accordingly.

We performed a detailed statistical analysis of the distribution of these therapies across the four NK cell dosing groups, with results summarized in the new Supplementary Table 2. We performed statistical analysis of concomitant therapies distribution across groups using Fisher's exact test. The results showed no significant differences in TACE (P=0.65), RFA (P=0.50), apatinib (P=0.70) or surgical intervention (P=0.17) distribution, suggesting balanced baseline characteristics. Although sorafenib use differed significantly among groups (P=0.02), the cohorts with the best survival outcomes (Group C, 33.3%; Group D, 75%) did not correspond to those with the highest administration rates. This indicates that the survival benefit cannot be attributed solely to sorafenib exposure. We have incorporated this important observation into the Results section (Page 11-12, Lines 240-246),

**S****upplementary Table 2. Distribution of Concomitant Anticancer Therapies Across NK Cell Dosing Groups**

| **Concomitant Therapy Modality** | **Group A**  **(n=3)** | **Group B**  **(n=5)** | **Group C**  **(n=6)** | **Group D**  **(n=4)** | **Overall**  **(n=18)** | ***P*-Value** |
| --- | --- | --- | --- | --- | --- | --- |
| **Loco-regional Therapy** |  |  |  |  |  |  |
| TACE | 0 (0.0%) | 2(40.0%) | 1( 16.7%) | 2(50%) | 5(27.8%) | 0.65 |
| RFA | 0(0%) | 0 (0%) | 1(16.7%) | 1(25%) | 2 (11.1%) | 0.50 |
| **Systemic Therapy** |  |  |  |  |  |  |
| Sorafenib | 3(100.0%) | 0(0.0%) | 2 (33.3%) | 3(75%) | 8 (44.4%) | 0.02 |
| Apatinib | 0 (0.0%) | 2(40%) | 2 (33.3%) | 1(25%) | 5 (27.8%) | 0.70 |
| **Surgical Intervention** |  |  |  |  |  |  |
| Surgical Resection | 1 (33.3%) | 0(0%) | 0 (0%) | 0 (0%) | 1 (5.6%) | 0.17 |

*Data are presented as number of patients (percentage) unless otherwise specified. P-values for categorical variables (TACE, Radiotherapy, Sorafenib, Apatinib, Surgical Resection) were calculated by Fisher’s exact test. TACE: transcatheter arterial chemoembolization, RFA: radiofrequency ablation,

In the Limitations section, we now state: "The small, non-randomized sample size and the heterogeneity of concomitant anticancer treatments received by patients are considerable limitations that preclude definitive conclusions on clinical efficacy. Although our post hoc analysis indicated no significant differences in the distribution of major concomitant therapies across groups, we cannot entirely rule out their potential influence as confounding factors.” (Page 20-21, Lines 439-443),
